# Mutations in the circadian cycle drive adaptive plasticity in cyanobacteria

**DOI:** 10.1073/pnas.2506928122

**Published:** 2025-09-03

**Authors:** Alfonso Mendaña, María Santos-Merino, Raquel Gutiérrez-Lanza, Marina Domínguez-Quintero, Juan Manuel Medina-Méndez, Ana González-Guerra, Víctor Campa, Miguel Baez, Magaly Ducos-Galand, Rocío López-Igual, Daniel C. Volke, Muriel Gugger, Pablo I. Nikel, Didier Mazel, Fernando de la Cruz, Raúl Fernández-López

**Affiliations:** ^a^Instituto de Biomedicina y Biotecnología de Cantabria, Universidad de Cantabria—Consejo Superior de Investigaciones Científicas, Santander, Cantabria 39011, Spain; ^b^Institut Pasteur, Université Paris Cité, CNRS UMR3525, Unité Plasticité du Génome Bactérien, Paris 75015, France; ^c^The Novo Nordisk Foundation Center for Biosustainability, Technical University of Denmark, Kongens, Lyngby 2800, Denmark; ^d^Institut Pasteur, Université Paris Cité, Collection of Cyanobacteria, Paris 75015, France

**Keywords:** Cyanobacteria, circadian rhythm, experimental evolution

## Abstract

Circadian clocks allow organisms to anticipate daily fluctuations in light and temperature, but how this anticipatory role promotes adaptation to different environments remains poorly understood. Here, we subjected the cyanobacterium *Synechococcus elongatus* PCC 7942 to a long-term evolution experiment under high light, high temperature, and elevated CO_2_ levels. After 1,200 generations, we obtained a strain exhibiting a 600% increase in growth rate. Whole-genome sequencing revealed three mutations fixed in the evolved population, two of which were sufficient to recapitulate the fast-growing phenotype in the wild type. A mutation in the promoter of the shikimate kinase *aroK* led to its overexpression, while a mutation in the central circadian regulator *sasA* disrupted both the phase and amplitude of the circadian rhythm. Changes in circadian control led to widespread perturbations in the transcriptome and metabolome. These included major shifts in the Calvin–Benson–Bassham cycle and glycogen storage dynamics. While these changes increased fitness under the experimental conditions, they caused maladaptation when light or CO_2_ levels were altered, revealing a trade-off between fitness and environmental flexibility. Our results demonstrate that mutations in circadian control can drive fast adaptation by modulating central metabolism, underscoring the circadian cycle as a cornerstone of cellular plasticity. Thus, targeting the circadian cycle could be key to engineering cyanobacterial strains optimized for carbon fixation and biomass production.

## Significance

In most organisms, circadian clocks regulate day and night-time physiology. The ubiquity of circadian regulation suggests it provides some adaptive advantage, but its contribution to fitness remains unclear. In cyanobacteria, most genes are regulated in a circadian fashion. These photosynthetic microorganisms also display a surprising phenotypic plasticity, with members of the same species showing large differences in growth rates. To study the interplay between the circadian rhythm and fitness, we performed a long-term evolution experiment where cyanobacteria were grown under continuous light for 1,200 generations. Mutations in circadian control produced a strain with a massive gain in fitness, revealing that circadian regulation facilitates evolvability. Engineering the clock may thus be key for the generation of fast-growing strains for biotechnological applications.

Initially considered to be too simple to sustain a circadian clock, cyanobacteria have emerged as one of the best-studied models of circadian regulation. Key players in the carbon and oxygen cycles on earth, cyanobacteria employ photosynthesis to produce ATP and reducing power. To avoid the generation of lethal reactive oxygen species, cyanobacteria must carefully balance their metabolism with external light, thus their physiology is heavily influenced by circadian rhythms (1). The cyanobacterial clock is based on a posttranscriptional oscillator (PTO) formed by the products of the *kaiABC* cluster (1–5). Fluctuations in the PTO are converted into transcriptional cycles through RpaA, the master transcriptional regulator of the circadian cycle (6, 7). Genes controlled by RpaA can be divided into two classes (8). Class I genes are activated by RpaA and achieve a maximum at dusk, while Class II are repressed by RpaA and peak at dawn (6, 7, 9). In the model cyanobacterium *Synechococcus elongatus* PCC 7942 (hereafter PCC 7942), 64% of the genes are under circadian transcriptional regulation (8, 10).

Maintaining energy levels through the regulation of glycogen metabolism has been the classical function assigned to the clock (11–13). However, in PCC 7942, a wide range of physiological functions, such as stress responses, natural competence, and cell division, are regulated in a circadian fashion, suggesting that the clock may play roles beyond energetic balance (14–18). Despite our knowledge of its molecular mechanisms, the ecological role of circadian regulation remains elusive. While the core components of the clock are highly conserved across cyanobacteria, its physiological relevance varies between species (19, 20). Marine picocyanobacteria, such as *Prochlorococcus* spp., contain a simpler mechanism lacking key features of a bona fide clock (21). Other model species, like *Synechocystis* sp. PCC 6803, exhibit less marked circadian oscillations (22), and in *Cyanothece* sp. ATCC 51142, ultradian rhythms longer than 24 h have been observed (23). Overall, the reasons for these differences are unclear. It has been shown that similar cyanobacteria exhibit substantial divergence in growth rates and environmental preferences, even among isolates of the same species (24). In *S. elongatus*, for example, model strains like PCC 7942 and PCC 6301 tolerate moderate light intensities and show optimal doubling times of approximately 6 to 8 h. Yet, *S. elongatus* UTEX 2973 (hereafter UTEX 2973), a *S. elongatus* strain that is 99.99% identical at the DNA level to PCC 7942, grows optimally at much higher light intensities, with doubling times as short as 2 h (25).

To identify the genetic basis of this phenotypic plasticity, we subjected PCC 7942 to a long-term evolution (LTE) experiment. The strain was grown under continuous, high intensity illumination, high temperature, and high CO_2_ for 2 y, which corresponded to 1,200 generations. At the end of the LTE, the evolved population grew six times faster than its ancestral counterpart. Genetic, transcriptomic, and metabolomic analyses revealed that adaptation to fast growth involved profound alterations in the circadian cycle. A comparison between the transcriptomes of our evolved strain and UTEX 2973, another fast-growing isolate, showed that both strains adapted to growth under high light intensities through convergent transcriptional strategies, yet the driving mutations were different. Altogether, our results demonstrate that mutations in circadian control have drastic effects on the cyanobacterial phenotype and are key for *S. elongatus* to adapt to different environmental regimes.

## Results

### LTE.

**Fig. 1.**
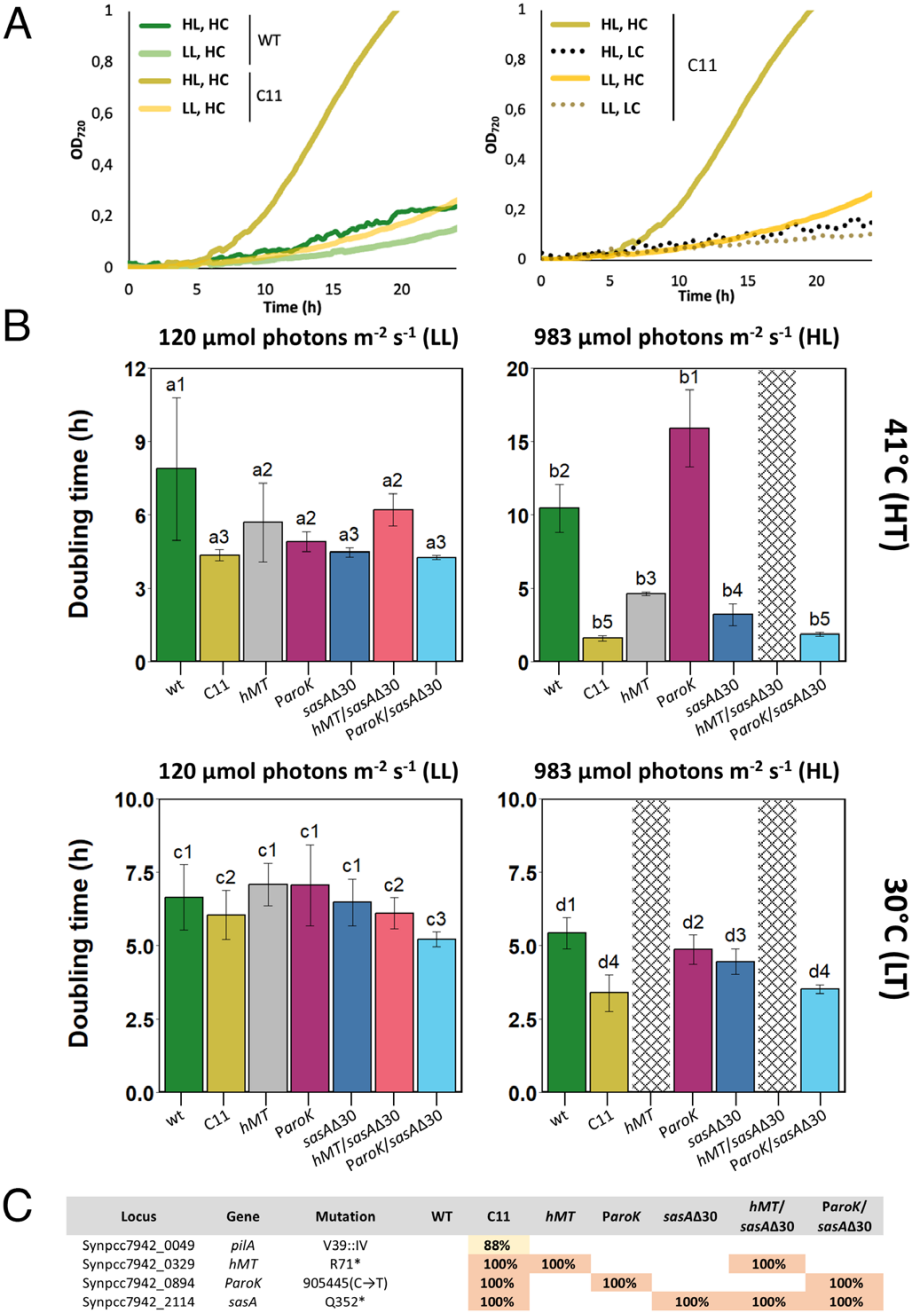
Growth rates of *S. elongatus* PCC 7942 ancestral and evolved strains in different conditions. (*A*) Growth curves of the ancestral (wt) and evolved (C11) strains monitored as the OD_720_ (y-axis) along time (x-axis). The panel on the *Left* shows the growth advantage of C11 at high light intensity (HL, 983 µmol photons m^−2^ s^−1^, dark yellow line) and high CO_2_ (HC, 3% CO_2_) compared to the growth curves of C11 at moderately low light intensity (LL, 120 µmol photons m^−2^ s^−1^ light yellow line) and the wt at HL (dark green line) and LL (light green line) intensities. All growth curves on this panel were carried out at 41 °C (HT) and 3% CO_2_ (HC) saturation. The right panel shows the growth advantage at HL intensity (dark yellow line) disappearing when the culture is grown at atmospheric CO_2_ concentrations (0.04%, LC; dashed lines). (*B*) Growth rates, expressed as the doubling time, of C11, wt, and point mutants at different temperatures (41 °C, HT, upper; 30 °C, LT, lower) and light intensities (120 µmol photons m^−2^ s^−1^, LL, left; 983 µmol photons m^−2^ s^−1^, HL, *Right*). The growth curves to calculate these growth rates were carried out at 3% CO_2_ (HC) under continuous illumination. Bars indicate the mean and SD of at least four independent replicates. Dashed bars represent conditions for which no growth was observed. Bars under the same letter-number combination do not show statistically significant differences (*P* > 0.05 by Tukey´s multiple comparison test). Bars under different letter-number combinations do show statistically significant differences by the same metric. (*C*) Location of the mutations fixed or nearly fixed (>88% reads) in C11. Percentages indicate the fixation index of each mutation in the evolved strain (C11) and point mutants, named after their respective mutations.

PCC 7942, a strain with a planktonic phenotype, incapable of forming biofilms, was chosen for a LTE experiment under serial passage (26). A master culture of PCC 7942 was retrieved from the Pasteur Cultures of Cyanobacteria (PCC) collection to serve as the initial population. During the evolution experiment, cells were grown in BG11 medium at 41 °C, under continuous illumination at 1,313 µmol photons m^−2^ s^−1^, and constant bubbling of 5% CO_2_. Culture dilutions were performed every six to seven generations, starting with a 1:130 dilution that ensured an effective population size of at least 10^9^ cells. After 1,248 generations, we obtained an evolved strain, named C11, which reached saturation significantly faster than its ancestral counterpart. Analysis of the pigment content of C11 revealed a statistically significant increase in chlorophyll *a* (Chl*a*), which was 14% increased in C11, compared to the wt (*SI Appendix*, Fig. S1). However, when we compared the colony-forming units (CFUs) obtained per unit of optical density measured at 720 nm unit (OD_720_), no significant differences between the evolved and ancestral strains were observed (*SI Appendix*, Fig. S1). This allowed us to compare the growth rates of both strains by tracking OD_720_ over time (Fig. 1). Growth curves in batch cultures showed that, under conditions of high light intensity (HL, 983 µmol photons m^−2^ s^−1^), high temperature (HT, 41 °C), and high CO_2_ levels (HC, 3%), the generation time of C11 was 1.6 h, representing a 654% increase compared to the wild-type (wt) (Fig. 1 *A*, *Left*). This fitness gain, however, depended on the specific environmental conditions. Reducing the illumination to 120 µmol photons m^−2^ s^−1^ (LL) or lowering the temperature to 30 °C (LT) resulted in a decrease in the fitness of C11 (Fig. 1*B*). The effect of light intensity was most pronounced at HC, where a reduction from 983 to 120 µmol photons m^−2^ s^−1^ led to a drastic drop in the growth rate (Fig. 1*A*). Overall, carbon availability was the most critical environmental factor. When C11 was grown under atmospheric CO_2_ concentrations (LC, 0.04%), its growth rate decreased sixfold, regardless of light intensity (Fig. 1 *A*, *Right*). As a result, at low CO_2_ levels the wt grew faster than C11 in all conditions tested (*SI Appendix*, Fig. S2). Altogether, these results indicate that the adaptation to the physical environment of the experiment (HL, HT, HC), resulted in a maladaptive phenotype in different ecological conditions.

### Reconstruction of the Phenotype in the Wild Type.

To identify the causes of adaptation, we sequenced the genome of C11 and compared it with its ancestral strain. A frozen sample of the evolving population obtained at generation 824 (G824) was also introduced in the analysis to follow evolutionary trajectories (Dataset S1). When the genomes of C11 and PCC 7942 were compared, we identified four mutations that were largely fixed (present in >80% of the reads) in C11 (Fig. 1*C*). A mutation in *pilA1* (Synpcc7942_0048), a gene required for natural competence in cyanobacteria, was found in 88% of the C11 population. This mutation caused loss of transformability (*SI Appendix*, Fig. S3), a phenotype often observed in model cyanobacterial strains under laboratory cultivation (24, 27). Three additional mutations were fixed in the C11 population. The first one caused an early *STOP* codon in the gene Synpcc7942_0329, encoding a putative class I SAM-dependent methyl transferase (Refseq: WP_011377536). We named this mutation *hMT*, hypothetical Methyl Transferase. Homologs of this protein in *Synechocystis* sp. PCC 6803 and *Synechococcus* sp. PCC 7002 (CpcM) have been involved in the posttranslational methylation of phycobiliproteins, contributing to the efficiency of energy transfer in the photosynthetic chain (28). This mutation truncated the polypeptide at R71 which, given the length of the wt protein, is likely to result in loss of function. The second mutation (P*_aroK_*) involves a C→T transition in the promoter region of *aroK* (Synpcc7942_0894, P*_aroK_*), a gene encoding shikimate kinase, which catalyzes a key phosphorylation reaction in the chorismate synthesis pathway (29). Finally, the third mutation (*sasA*Δ30) introduces a premature *STOP* codon in *sasA* (Synpcc7942_2114), a gene involved in circadian regulation. SasA is a histidine-kinase that phosphorylates RpaA, the master regulator of circadian promoters (6, 30). The *sasA*Δ30 mutation in C11 truncates SasA at Q352, leading to a 30-amino acid deletion at the C-terminus, which contains the histidine kinase domain. Among these mutations, both P*_aroK_* and *sasA*Δ30 were present in the population at G842 (Dataset S1), whereas *hMT* emerged later in the evolutionary process.

To test the impact of these mutations, we introduced them in the genome of PCC 7942, replacing wt alleles with their mutant counterparts. The growth rate of each individual mutant was measured under various environmental conditions and compared to the wt (Fig. 1*B*). The P*_aroK_* mutation alone did not improve growth in any of the conditions tested. Both *sasA*Δ30 and *hMT* outperformed the WT under LTE conditions but grew significantly slower than C11. These results show that, in isolation, none of the mutations recapitulated the phenotype of C11. We thus introduced combinations of mutations in the genome of the wt. As shown in Fig. 1*B*, the combination of P*_aroK_* and *sasA*Δ30 resulted in growth rates equivalent to that of C11. As it was the case for the C11 isolate, the growth advantage brought about by the P*_aroK_* and *sasA*Δ30 mutations was only observed in HL, HT, and HC conditions. All our attempts at producing a triple mutant (*hMT*, P*_aroK_, sasA*Δ30) under standard conditions for genetic transformation (31) were unsuccessful, and the *hMT* mutation proved lethal in HL and LT (Fig. 1*B*), a phenotype previously reported for its homolog in *Synechocystis* (28). These results thus indicate that the P*_aroK_* and *sasA*Δ30 mutations are key for recapitulating the fast-growth phenotype of the evolved strain. A two-way ANOVA test for the main effects and interactions of environmental conditions and mutations revealed that C11, *sasA*Δ30, and the P*_aroK_*/*sasA*Δ30 double mutant globally improved their growth rate as the light intensity increased. In contrast, increasing light and temperature led the wt and the P*_aroK_*to lower growth rates, thus underscoring the specific adaptation of C11 to the LTE conditions (*SI Appendix*, Fig. S4 and Table S1).

### The Evolved Strain Is Locked in a Transcriptional Response to High Light.

**Fig. 2.**
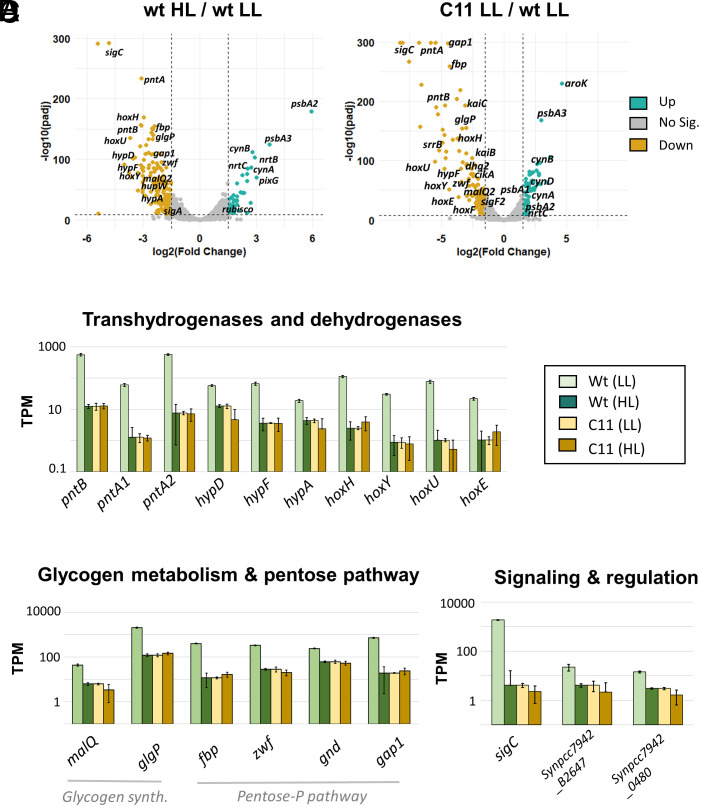
C11 transcriptome is locked in a high-light response mode. (*A*) Volcano plots showing the changes in gene expression, indicated as log_2_ of the fold change in TPMs (transcripts per million), in the wt and C11 at 30 °C, HC, and different light intensities. Dots represent the log_2_ fold change (x-axis) against its adjusted *P*-value (y-axis). Dots shown in teal correspond to genes with at least 1.5-fold increase with adjusted *P*-value < 10^−8^. Dots shown in orange correspond to genes with 1.5-fold decrease in expression with adjusted *P*-value < 10^−8^. The leftmost graph corresponds to fold changes experienced by the wt in moderately low light (LL, 120 µmol photons m^−2^ s^−1^) compared to high light (HL, 983 µmol photons m^−2^ s^−1^). The rightmost graph corresponds to the changes observed in C11 compared to wt when both strains were grown under LL intensities. (*B*–*D*) Transcriptional levels, expressed as the average TPMs and the SD of three independent replicates, of genes repressed in HL in the wild type, which are constitutively downregulated in C11. Genes shown include hydrogenases (*B*), glycogen synthesis and the pentose phosphate pathway (*C*), and signaling and regulation (*D*).

To investigate the causes of the fast-growth phenotype, we conducted comparative transcriptomic analyses of C11 and the wt under high light and low light conditions (Dataset S2). We first identified genes that were differentially expressed in response to high-light intensity. For this purpose, we measured the transcription levels in the wt when grown at 983 µmol photons m^−2^ s^−1^ (HL), and 120 µmol photons m^−2^ s^−1^ (LL). As shown in the volcano plot in Fig. 2*A* (*Left* panel), among the genes with increased transcription, we identified well-known high-light-responsive genes, such as *psbA2*, which encodes the D1 protein of photosystem II. Genes downregulated by HL in the wt included the sigma factor *sigC*, several hydrogenase-related genes (*hox*, *pnt*, *hyp*), and genes involved in glycogen metabolism and the oxidative pentose phosphate (oxPP) pathway.

We then compared the transcriptomes of C11 and the wt in LL, and the volcano plot of this comparison (Fig. 2 *A*, *Right* panel) was surprisingly similar to the previous one (Fig. 2 *A*, *Left* panel). This indicated that C11 showed a transcriptional profile equivalent to that of the wt grown under HL. To clarify this observation, we also measured the transcriptomes of C11 and the wt in HL (*SI Appendix*, Fig. S5). A comparison of the transcription levels between the evolved and the ancestral strain under HL and LL revealed that genes downregulated by HL in the wt were constitutively repressed in C11 (Fig. 2 *B*–*D*). Similarly, genes upregulated by HL in the wt were also constitutively overexpressed in the evolved strain (*SI Appendix*, Fig. S6). Thus, the transcriptome of C11 mirrors that of the wt exposed to high-light intensities.

**Fig. 3.**
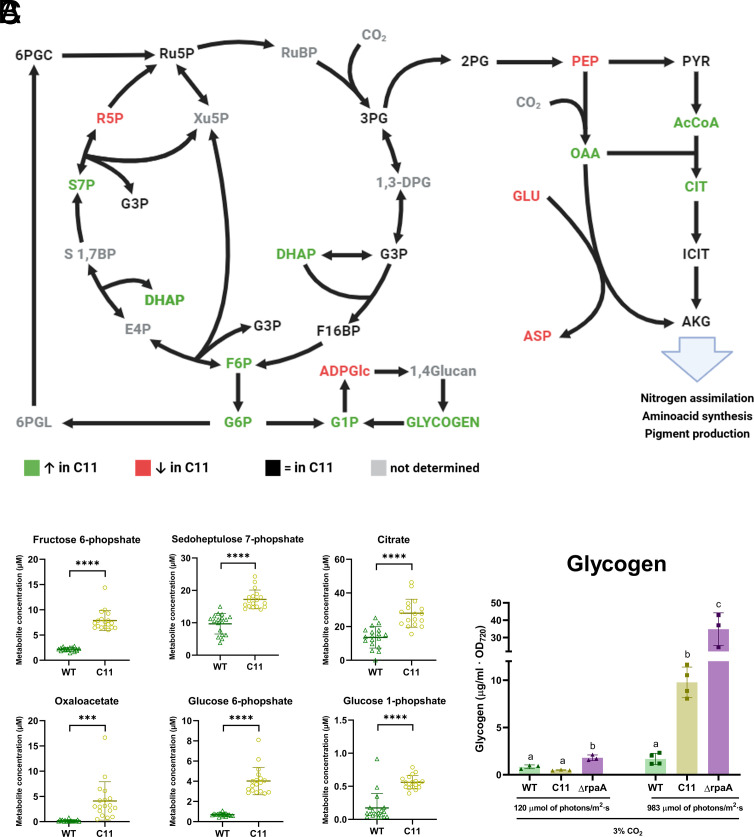
Alterations in central carbon pathways in the evolved strain. (*A*) Diagram showing the key metabolites of central carbon metabolism pathways, including the CBB (*Left*), the Tricarboxylic Acid cycle (*Right*) and the glycogen synthesis pathway (*Bottom*). Metabolites in green were found significantly increased (*P*-value < 0.05) in C11. Metabolites in red were found significantly decreased in C11 (*P*-value < 0.05). No significant changes were found in metabolites shown in black. Metabolites shown in gray were not measured. All measurements were performed at 30 °C, under HL and HC conditions. (*B*) Concentrations of selected metabolites (y-axis) in the wt (green) and C11 (yellow). The chart shows the levels of the six metabolites with the most significant increase in C11. ***P-value < 0.001, ****P-value < 0.0001. All measurements were performed at 30 °C, under HL and HC conditions. (*C*) Glycogen levels, expressed as µg mL^−1^ normalized by the OD_720_ of the wt (green) and C11 (yellow) and a Δ*rpaA* circadian mutant (purple). Each bar represents the average of three biological replicates. Bars with the same letter within the same condition do not differ statistically (*P*-value > 0.05) by Tukey’s multiple comparison test. All measurements were performed at 30 °C and HC conditions, under the indicated light intensities.

Many of the genes transcriptionally altered in C11 are involved in key metabolic pathways. For this reason, we carried out a metabolomic comparison between C11 and the wt under LL conditions. Results, summarized in Fig. 3, and detailed in *SI Appendix*, Fig. S7 and Dataset S3, demonstrate that C11 exhibited increased levels of several intermediates of the Calvin–Benson–Bassham cycle (CBB), including fructose 6-phosphate and sedoheptulose 7-phosphate (Fig. 3*B*). Anaplerotic carbon fixation into oxaloacetate was also apparently enhanced in C11, as judged from higher oxaloacetate levels (Fig. 3*B*) along with increased transcription of the PEPC enzyme (Dataset S2). Taken together, these results pointed to an increased carbon fixation rate in C11, which would explain its faster growth rate. A global shift toward anabolic metabolism was also apparent in the expression of the two isoforms of fructose 1,6-bisphosphatase (*fbp* and *fbpI*). C11 showed a downregulation of *fbp*, and a moderate increase in *fbpI*, which is required to sustain photosynthesis and gluconeogenesis in *Synechococcus* (32). Similarly, the *gap1* gene, encoding an NAD^+^-dependent glyceraldehyde-3-phosphate dehydrogenase, was downregulated in strain C11, while the expression of the *gap2* gene, an NADP^+^-dependent isoform, remained unchanged (Dataset S2). In *Synechocystis* sp. PCC 6803 and other eubacteria, *gap1* is involved in catabolism, whereas *gap2* has anabolic functions (33).

Increased carbon fixation and rerouting of metabolism toward anabolism were accompanied by other shifts in central carbon and energy metabolism. In C11, we observed high levels of glucose 1-phosphate and glucose 6-phosphate (Fig. 3*B*). The evolved strain also presented higher glycogen levels than the wt strain under a high carbon atmosphere (Fig. 3*C*). These metabolomic changes correlated with the downregulation of genes involved in the oxPP pathway and glycogen mobilization (Fig. 2*C*), suggestive of a substantial rerouting of carbon and storage compound fluxes in the evolved strain. Interestingly, similar changes in glycogen metabolism and the oxPP pathway were observed in a Δ*rpaA* strain, linking perturbations in circadian regulation to the phenotype of C11.

### C11 Presents Perturbations in the Circadian Cycle.

**Fig. 4.**
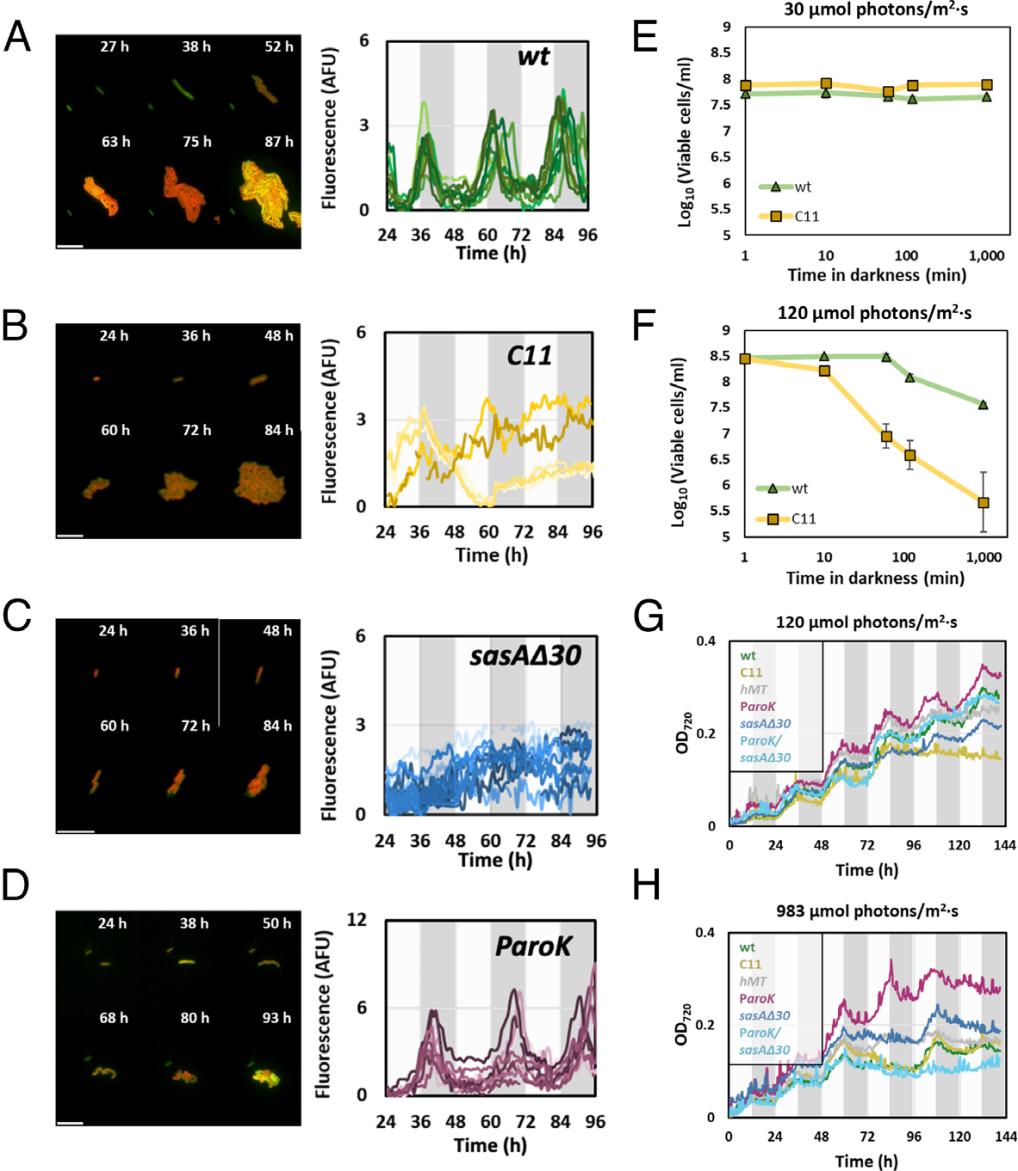
C11 presents perturbations in the circadian cycle. (*A*–*D*). Microphotographs (*Left*) and fluorescence traces (*Right*) obtained from a P*_sigC_*-YFP transcriptional fusion in each of the strains indicated. Time-lapse experiments were performed on cultures presynchronized after growing for 72 h in LD conditions and then subjected to continuous light. Each trace on the graphs represents the values obtained for a single cell, tracked for 72 h under continuous illumination. Dark and light areas of the charts indicate, respectively, the periods of subjective night and day, that is, the phases for which the circadian clock of the cyanobacteria anticipates darkness or light, regardless of the actual external illumination. All experiments were performed under LC and LT conditions, and 60 µmol photons m^−2^ s^−1^. Scale bar, 10 µm. (*E* and *F*) Darkness-induced lethality. Cultures of the wt (green) and evolved (yellow) strains were grown under continuous very low (*E*, 30 µmol photons m^−2^ s^−1^) or low (*F*, 120 µmol photons m^−2^ s^−1^) light intensities and exposed to total darkness for a period indicated in the x-axis. The number of viable cells after darkness exposure (y-axis) was determined by plating, as indicated in materials and methods. Each data point represents the average and SD of three independent experiments. (*G* and *H*) Growth curves, monitored as OD_720_ (y-axis) along time (x-axis) under light–dark conditions. Cultures were grown under 0.04% CO_2_ (LC) concentrations, 30 °C, and a regime of 12 h light + 12 h darkness (gray areas). Light intensity was set at 120 µmol photons m^−2^ s^−1^ (*G*) or 983 µmol photons m^−2^ s^−1^ (*H*).

Whole-genome sequencing revealed a *sasA*Δ30 mutation present in C11, which caused a deletion in the kinase domain of SasA. SasA is the protein that phosphorylates the circadian regulator RpaA to its active conformation. Since an active RpaA is required to sustain circadian transcription, the *sasA*Δ30 mutation could potentially disrupt the circadian rhythm of C11. To test this possibility, we measured the expression of *sigC*, a prototypical Class I gene (9, 34). For this purpose, a yellow fluorescent protein (YFP_LVA) was transcriptionally fused to the *sigC* promoter P*_sigC_* (34). This transcriptional reporter was introduced into the NS1 neutral site of the wt, C11, *sasA*Δ30, P*_aroK_*, and *hMT* strains. P*_sigC_* expression profiles were obtained by measuring YFP levels in single cells using time-lapse microscopy (Fig. 4 and Movies S1–S4). In the wt, P*_sigC_* showed the fluorescence profile expected of a prototypic Class I promoter (Fig. 4*A*), with a peak at dusk, a trough at dawn, and a circadian period of 23.8 ± 0 h (*SI Appendix*, Fig. S8). In contrast, P*_sigC_* expression in C11 was arrhythmic (Fig. 4*B*). To identify the mutation driving this phenotype, we analyzed the expression profile of P*_sigC_* in the different point mutants. P*_sigC_* activity in the P*_aroK_* mutant background showed a robust circadian behavior (Fig. 4*D*), with a period of 23.5 ± 0 h (*SI Appendix*, Fig. S9). The period in this mutant was thus similar to the wt, although the amplitude of the circadian fluctuation in P*_sigC_* increased 3-fold. P*_sigC_* circadian fluctuations in the *hMT* mutant maintained the amplitude of the wt but displayed a slightly shorter period of 21.4 ± 5.316 h (*SI Appendix*, Figs. S10 and S11). P*_sigC_* activity in the *sasA*Δ30 mutant showed an arrhythmic phenotype, similar to C11 (Fig. 4*C*). Results thus indicated that this mutation is key to the loss of circadian expression in the evolved strain. The defect in C11 rhythmicity was confirmed using a P*_psbA1_*-YFP construct, which yielded equivalent results in the first 48 h of the experiment. (*SI Appendix*, Fig. S12). Overall, results showed that C11 displayed an abnormal circadian cycle, with *sasA*Δ30 as the driving mutation.

Deletions of *sasA* or *rpaA* have been shown to impair growth in light–dark cycles (LD) and cause darkness-induced lethality, a phenotype where cells die after being exposed to short periods of total darkness (6, 30, 35). We thus measured the growth rates of C11, the wt, and the individual mutants in diel cycles (12 h light/12 h darkness), varying the light intensity during daytime. Cycles of 12 h light/12 h darkness at 41 °C and LC were found to be lethal for the wt, thus measurements were performed at 30 °C. Under these conditions, when we grew cells in a LD cycle characterized by a moderate diurnal light intensity (120 µmol photons m^−2^ s^−1^), C11 and *sasA*Δ30 showed decreased growth rates after 72 h (Fig. 4*G*). Interestingly, the strain carrying the P*_aroK_* mutation grew faster than the wt, and, when introduced into a *sasA*Δ30 background, this mutation restored the growth rate to wt levels. The advantage provided by the P*_aroK_* mutation was most obvious when the LD conditions included a strong daylight intensity (983 µmol photons m^−2^ s^−1^). Under these conditions, even the wt arrested growth after 72 h, while the P*_aroK_* mutant was able to keep growing at the fastest pace (Fig. 4*H*). Both C11 and the double mutant P*_aroK_/sasA*Δ30 also suffered from arrested growth in these conditions.

To test whether the impaired growth of C11 in LD cycles was due to darkness-induced lethality, as in a Δ*rpaA* mutant (15), we measured cell viability after a period of total darkness. For this purpose, we grew cells under continuous light, exposing them to a period of total darkness of variable duration, and then measured the viability of the culture. As shown in Fig. 4*E*, we observed no darkness-induced lethality when cells were grown at very low light intensity (30 µmol photons m^−2^ s^−1^), as previously reported for the Δ*rpaA* strain (15). When cells were grown at 120 µmol photons m^−2^ s^−1^, however, C11 showed a sharp decrease in viability after 60 min of darkness (Fig. 4*F*). Our results thus indicated that C11 presents levels of darkness sensitivity similar to a Δ*rpaA* mutant.

### Gene Expression in C11 Is Correlated to a Δ*rpaA* Mutant and UTEX 2973.

**Fig. 5.**
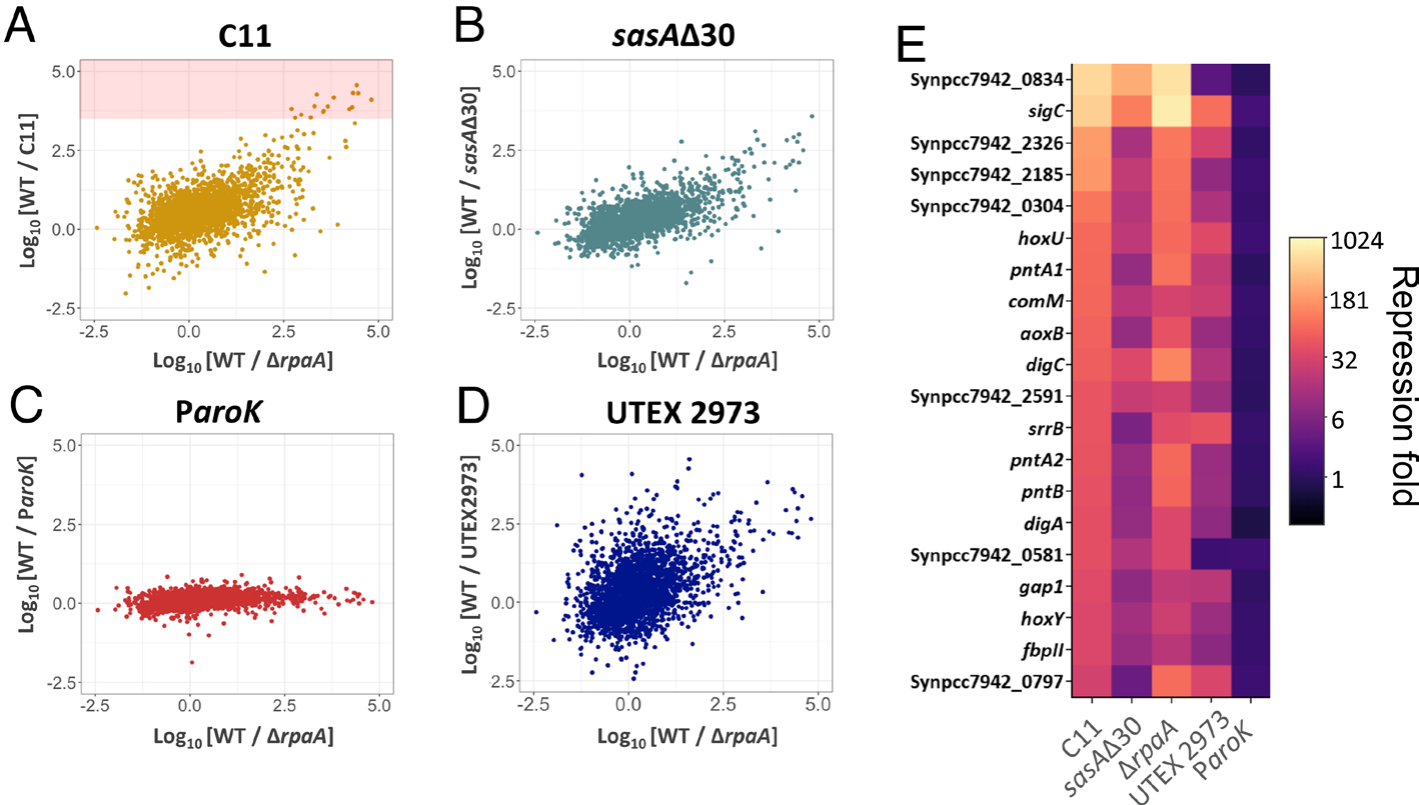
Gene expression in C11 is correlated to a *ΔrpaA* mutant and UTEX 2973. (*A*–*D*) Scatterplots showing the repression fold, expressed as the Log_10_ of the TPMs exhibited by a particular gene in the wt, divided by the corresponding mutant (y-axis), compared to those of Δ*rpaA* (x-axis). The red area in panel 5A represents the levels corresponding to the 20 most repressed genes in C11. Cultures were grown at 30 °C, HC, and HL conditions. (*E*) Heatmap showing the repression fold, calculated as before, and expressed in the color chart shown in the legend, for the 20 most repressed genes in C11, in each of the mutants shown in the x-axis of the figure.

C11 showed some hallmarks of a Δ*rpaA* strain, such as an arrhythmic expression in P*_sigC_* and P*_psbA1_*, darkness-induced lethality, and the repression of genes involved in glycogen metabolism and the oxPP pathway. For these reasons, we compared the transcriptomes of C11 (and each of the point mutants) with that of a Δ*rpaA* strain. We calculated the fold-change values with respect to the wt, and we plotted them against the fold-change values of a Δ*rpaA* strain (Fig. 5 *A*–*D*). In the case of the P*_aroK_* mutant strain (Fig. 5*C*), values scattered along a flat line across the x-axis, indicating no correlation with Δ*rpaA* (Pearson’s correlation coefficient, r = 0.06). However, a positive correlation was observed in C11 (r = 0.89) and *sasA*Δ30 (r = 0.71), as shown in Fig. 5 *B* and *C*. The transcription of genes highly repressed in these strains was also repressed in a Δ*rpaA* background. All 20 genes that showed the highest downregulation in C11 and *sasA*Δ30 were repressed by at least fivefold in Δ*rpaA* (Fig. 5*E*). Altogether, these results indicate that the *sasA*Δ30 mutation caused transcriptomic changes similar to that observed in a Δ*rpaA* mutant, suggesting a perturbed SasA-RpaA transduction pathway in *sasA*Δ30 and C11.

Intriguingly, UTEX 2973, a strain that, like C11, grows significantly faster under HL conditions, also harbors mutations in RpaA that are essential for its phenotype (25). We thus took advantage of the transcriptomic data available for UTEX 2973, and compared it, as in the previous case, with that of a Δ*rpaA* mutant. As shown in Fig. 5*D*, we found a weak positive correlation (r = 0.25), but significantly lower than the correlation levels observed between Δ*rpaA* and C11 (r = 0.89) or Δ*rpaA* and *sasA*Δ30 (r = 0.71). The correlation between UTEX 2973 and Δ*rpaA* was mostly driven by genes that are highly repressed in both strains with respect to the wt (upper right quadrant in panel *D*, Fig. 5). Indeed, a close inspection revealed a remarkable degree of overlap among the genes present in this upper right quadrant in the comparisons with C11, *sasA*Δ30, and UTEX 2973. This indicates that there is a common set of genes that are highly repressed in Δ*rpaA*, which are also downregulated in C11, *sasA*Δ30, and UTEX 2973 (Fig. 5*E*). These include the sigma factor *sigC*, the *hox* hydrogenases, and the *gap1* glyceraldehyde dehydrogenase.

### Changes in the Phase and Amplitude of Class I and Class II Genes.

**Fig. 6.**
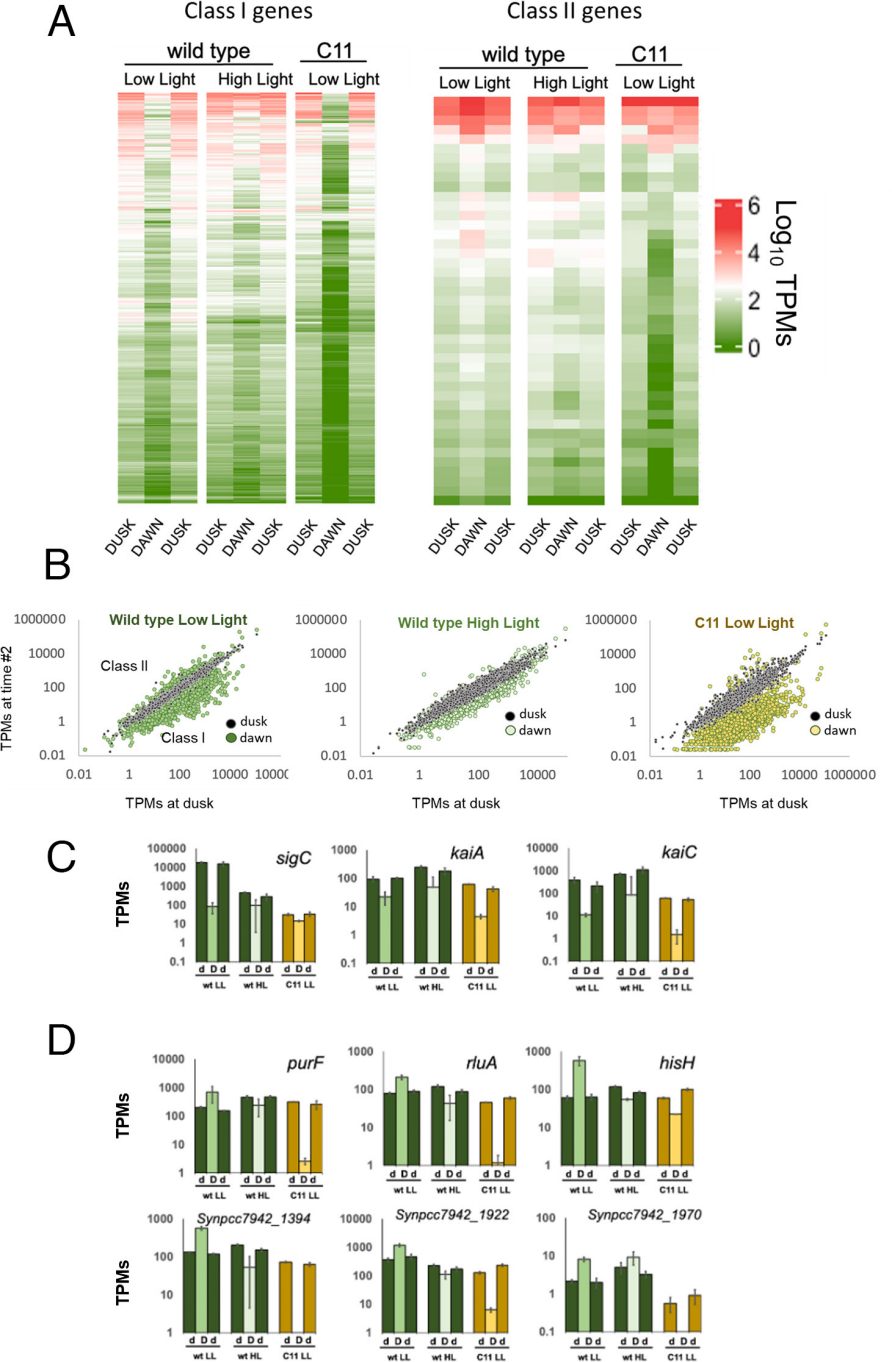
Changes in the phase and amplitude of class I and class II genes. (*A*) Heatmap showing the expression levels of Class I (*Left* panel) and Class II (*Right* panel) promoters, indicated as Log_10_ TPMs. All cultures were grown in a regime of 12 h day/12 h night cycles for four consecutive days, at 30 °C and HC conditions. The first column corresponds to the wt grown at a light intensity of 120 µmol photons m^−2^ s^−1^ (low light). The second column corresponds to the wt grown at a light intensity of 983 µmol photons m^−2^ s^−1^ (high light). The third column corresponds to C11 grown at a light intensity of 120 µmol photons m^−2^ s^−1^ (low light). For each column, the first lane corresponds to expression levels measured in the dusk of day 3, and the second and third lanes correspond, respectively, to the dawn and dusk of day 4. (*B*) Correlation of the expression levels of each gene at dawn and dusk. Cells were measured on the dusk of the first day (x-axis), dawn (colored dots), and the dusk of the second day (black dots). Expression levels measured at the two sequential dusk periods are expected to be similar, thus falling in the diagonal of the image. Class I genes show higher expression during dusk, and should appear below the diagonal, while Class II genes appear above. The charts correspond, from left to right, to values obtained in the wt grown at 120 µmol photons m^−2^ s^−1^ (LL), the wt grown at 983 µmol photons m^−2^ s^−1^ (HL), and C11 grown at 120 µmol photons m^−2^ s^−1^ (LL). (*C*) Transcription levels for selected Class I genes, including *sigC* (*Left*), *kaiA* (*Middle*), and *kaiC* (*Rightmost* panel). Bars correspond to transcription levels observed at dusk of day 3 (d), dawn of day 4 (D) and dusk of day 4 (d). Error bars indicate the SD. (*D*) Transcription levels for selected Class II genes. Bars and legend as in (*C*).

Since C11 exhibited traits similar to a Δ*rpaA* mutant, we studied gene expression levels across its entire genome in light–dark cycles. Under these conditions, the transcriptome of *Synechococcus* experiences the effects of the endogenous circadian cycle, as well as the impact of external fluctuations in light intensity during the day/night phases. These measurements are thus intended to identify the actual changes in expression experienced by C11, in comparison to the wt, when grown in physiological diel conditions. For this purpose, cells were grown in cycles of 12 h light/12 h darkness for four days, and total RNA was extracted at dusk of the third day, and the dawn and dusk of the fourth. We then performed a transcriptomic analysis, comparing gene expression levels at these three points (Dataset S4). In the wt, the expression of a majority of genes (2,159) exhibited a peak during dusk, thus behaving as Class I (Fig. 6*A*). A second set (428) showed a peak in transcription during dawn, characteristic of Class II genes. Finally, a total of 128 genes peaked neither at dusk nor dawn and were considered noncircadian. We restricted our analysis to genes with a strong circadian character, selecting those in which the amplitude of the fluctuation was higher than four SD of the average (*P*-value < 0.001). Using this criterion, we identified a total of 479 representative genes belonging to Class I and 43 genes belonging to Class II (Fig. 6*B*). When we analyzed these genes in C11, we found profound changes in their expression profiles. The most apparent was observed in Class II genes, which radically altered their phase. When we compared their profiles in the wt and C11 at low light intensities, we detected that most of the genes in this class have switched their phase (Fig. 6 *A*, *Right* panel). Instead of exhibiting a peak at dawn, as they do in the wt, Class II genes in C11 showed higher expression levels at dusk. This inversion of phase was observed in the entire class (Fig. 6*D*), affecting canonical genes such as *purF* (9) (Fig. 6*D*). As shown in the figure, this effect was caused by a repression of nighttime transcription, which pushed expression levels at dawn below those observed at dusk.

In C11, Class I genes also experienced decreased transcription during nighttime. However, since these genes are naturally downregulated during the night, the phase of this class was generally unaffected, and the most significant impact was an increase in the amplitude of the circadian fluctuation. We nevertheless identified a subset of Class I genes in which the downregulation of transcription also occurred during daytime, leading to the loss of their circadian character. Such was the case of *sigC* (Fig. 6*C*), confirming the results obtained in time-lapse experiments (Fig. 4*B*). Other classical Class I genes, such as *kaiA* or *kaiC* were, however, unaffected and maintained the phase and amplitude of their circadian fluctuation (Fig. 6*C*). These results thus demonstrate that, under physiological diel conditions, the circadian cycle is largely retained in C11, but the phasing and amplitude of many genes (especially of Class II) is significantly altered.

The circadian alteration in C11, together with the constitutive repression of genes downregulated by high light, suggested that light intensity during the day could somehow influence the phase and amplitude of the circadian rhythm in the wt. To study this possibility, we analyzed the impact of high intensity illumination during daytime in the circadian cycle of PCC 7942. We repeated the previous experiment, but in this case cells were grown in diel cycles in which the light intensity during daytime was increased to 983 µmol photons m^−2^ s^−1^. Results, shown in Fig. 6 *A* and *B*, indicated a global nighttime repression of all Class II genes, which again led to the reversal of their circadian phase. For Class I genes, however, expression levels during the night were elevated, leading to shallower fluctuations. In *sigC*, and the subset of genes that were downregulated and arrhythmic in C11, the circadian cycle was also abated. Thus, C11 presented some circadian perturbations that were also observed in wt cells exposed to high intensity illumination. The switching of Class II genes to a Class I phase, and the downregulation and loss of rhythmicity in *sigC* were the most conspicuous features.

## Discussion

Cyanobacteria are key primary producers in the biosphere, yet the environmental and genetic factors constraining their growth are poorly understood (36). Strains from the same species exhibit radically different reproductive rates and environmental preferences (25). To study the causes of this phenotypic diversity, we performed an LTE experiment growing the wt strain for ca. 1,200 generations under intense, continuous illumination. Direct evolution experiments have been used in cyanobacteria to achieve a number of desired phenotypes, including tolerance to toxic products (37, 38) and acclimation to high light and temperatures (39, 40). Despite their polyploid nature, cyanobacteria adapted fast to these environmental challenges. In our hands, the LTE experiment under HL, HC, and HT conditions resulted in a 600% increase in the growth rate in a relatively small number of generations. Compared to previous experiments with heterotrophic, haploid organisms such as *Escherichia coli* (41), the gain in fitness achieved in such a short time span was surprisingly high. This observation opened the possibility that the fast-growth phenotype observed in the evolved strain could be a wt feature of *S. elongatus* that was previously lost due to laboratory domestication. In PCC 7942, several phenotypic traits of *Synechococcus* wt, such as biofilm formation and phototaxis, were lost due to continued cultivation in the laboratory (42). Moreover, the recent isolation of UTEX 2973, a *S. elongatus* strain derived from UTEX 625 (42) that also grows significantly faster under HL and HC conditions, reinforced this possibility. Two lines of evidence, however, suggest otherwise. UTEX 2973 and C11 show no mutations in common, and genes essential for fast growth in C11 (such as *aroK*) are not mutated in UTEX 2973. On the other hand, recent isolates of *S. elongatus* grow at rates and environmental conditions similar to PCC 7942 and other legacy strains, suggesting that the fast-growth phenotype is not an ancestral trait lost during domestication (24). Like other freshwater β-cyanobacteria, PCC 7942 is primarily found in ponds and small water reservoirs prone to large and sudden environmental perturbations (43). In such contexts, a high phenotypic plasticity may contribute to cyanobacterial survival.

The fitness advantage observed in strain C11 was entirely dependent on the environmental conditions of the LTE (Fig. 1). When temperature, CO_2_ levels, or light intensity were altered, the fitness advantage of C11 disappeared. Under diel cycles, growth was significantly constrained (Fig. 4*G*), and darkness-induced lethality was evident (Fig. 4*F*). These findings indicate that the evolved strain experiences maladaptation under these conditions. Evolutionary trade-offs in the adaptation of species to differing environmental conditions are commonly observed (44). Two primary mechanisms are generally invoked to explain them: genetic drift, which leads to the accumulation of deleterious mutations, and antagonistic pleiotropy, where adaptive mutations in one environment are maladaptive in another (45). In our work, several lines of evidence suggest that both mechanisms may contribute to the trade-offs observed in the evolved strain. On the one hand, C11 exhibited large fitness gains in the LTE conditions, which were accompanied by pronounced deleterious effects under diel cycles. This observation is consistent with Fisher’s geometrical model, which predicts that larger mutational effects are more likely to negatively impact other traits, thereby exerting broad pleiotropic effects (46). On the other hand, the introduction of the *P_aroK_* and *sasAΔ30* mutations into the wild-type strain (wt) significantly improved fitness in the LTE conditions (Fig. 1) without imposing a substantial trade-off in diel cycles (Fig. 4*G*). This suggests that the trade-offs observed in C11 may not be solely attributed to antagonistic pleiotropy. Additionally, the fixation of nonadaptive mutations through genetic drift could also play a role. It is noteworthy that the evolved strain lost its natural transformation ability due to a mutation in *pilA*, which would drive the evolution of C11 to become asexual, thus favoring the fixation of epistatic mutations with neutral or negative impacts on fitness. Further research is required to elucidate the relative contributions of antagonistic pleiotropy and genetic drift in the adaptation of cyanobacteria to alternative environmental conditions.

In *Synechococcus*, the circadian cycle regulates most genes in the genome. Mutations perturbing the cycle are thus likely to have a broad impact on cellular physiology, potentially contributing to widespread pleiotropic effects. The master transcriptional regulator RpaA is a cornerstone of circadian control, and RNA sequencing revealed that the transcriptomic profiles of C11 and *sasA*Δ30 closely resembled that of a Δ*rpaA* mutant (Fig. 5). SasA is responsible for phosphorylating RpaA to its active conformation. Therefore, its C-terminal deletion may lead to an insufficiency in active RpaA levels, which would explain the similarities in transcriptomic profiles and the profound circadian perturbations observed in C11. Mutations in circadian control seem to be a requirement for the evolution of a fast-growth phenotype under continuous light, as UTEX 2973 also presents mutations in the SasA-RpaA axis that are essential for this phenotype. Suppressing the expression of Class II genes and the night metabolism may be beneficial in continuous light, as it has been previously hypothesized for cyanobacterial strains with no clock (47). Since the cycle is endogenously generated by the PTO, cells experience circadian transcription even when under perpetual daylight. Thus, abolishing the subjective night phase, a predominantly catabolic period when cell division is gated, may increase the overall growth rate. Data from C11 and UTEX 2973, however, indicate that while mutations in SasA-RpaA are necessary, they are not sufficient for a fast growth. In *Synechococcus* sp. PCC 7002, CRISPR interference against SasA and RpaA improved fitness under continuous light (48), but in PCC 7942 Δ*rpaA* mutants showed no advantage under continuous illumination (49–51). Recent experiments have shown that mutations in the circadian cycle boost protein production in PCC 7942 (52), thus suggesting that secondary mutations in C11 may be funneling the metabolic potential liberated by the abatement of night metabolism into effective biomass production (53). In C11, we observed increased levels of metabolic intermediates of the CBB cycle and anaplerotic pathways. Increased expression of *ppc*, an enzyme that directly carboxylates phosphoenolpyruvate using bicarbonate (54), could also contribute to the increased ability of strain C11 to fix CO_2_.

The role of the P*_aroK_* mutation in the phenotype of C11 may be related to stress tolerance. In PCC 7942 and *Synechocystis* sp. PCC 6803, mutations in *aroK* improve growth in high light and high temperature, but the exact molecular mechanism through which this mutation contributes to fitness remains uncertain (55). There are no mutations in the shikimate pathway in UTEX 2973, making it unlikely that both strains achieved the fast growth phenotype through identical mechanisms. This raises the question of whether combining the mutations of both strains could result in an additive effect. Such a strategy might enable the generation of faster-growing strains, serving as useful chassis for biotechnological and synthetic biology applications (56, 57).

In PCC 7942, fluctuations in the PTO are converted into a transcriptional oscillation through RpaA, but complex downstream regulation decomposes this initial tempo into transcriptional cycles of different phase and amplitude (35, 58). Our results demonstrate that light intensity specifically modulates these cycles, and that adaptation to high intensity illumination requires mutations in the circadian pacemaker. Previous work using computational models predicted that the fitness benefit conferred by the clock would decrease with growing light intensities (47). Our results support this prediction, suggesting, in turn, that the circadian rhythms of UTEX 2973 are probably perturbed too. Altogether, the phenotypic and transcriptomic characterization of C11 indicate that, in cyanobacteria, the circadian rhythm is not a mere instrument to separate day and night physiology. It is also an adaptive mechanism, providing phenotypic plasticity to ensure growth in different light intensities and environmental conditions.

## Materials and Methods

### Cyanobacterial Strains and Culture Growth Conditions.

*S. elongatus* PCC 7942 (PCC 7942) and all derived strains were routinely grown and maintained in liquid BG11 medium supplemented with appropriate antibiotics and 10 mM bicarbonate, when CO_2_ concentrations above atmospheric levels were used. Antibiotics used for selecting PCC 7942 were neomycin at 5 or 25 µg mL^−1^ (Neo5 or Neo25), spectinomycin at 10 or 20 µg mL^−1^ (Sp10 or Sp20) and streptomycin at 10 or 50 µg mL^−1^ (Sm10 or Sm50). Except otherwise stated, cells were grown under a continuous light flux of 60 µmol photons m^−2^ s^−1^ from white fluorescence lamps, in a Sanyo Plant Growth Chamber, at 30 °C and constant ambient air supply. Experiments with different light intensities, diel cycles, and CO_2_ concentrations were performed in a MC 1000-OD Multicultivator (Photon Systems Instruments), equipped with programmable temperature, CO_2_, and light intensity (cold white LED). Doubling times were determined from growth curves performed in a MC 1000-OD Multicultivator (Photon Systems Instruments). Growth was estimated from OD_720_ and OD_680_ measurements obtained every 10 min by the MC 1000-OD Multicultivator. The effective growth rate (K′) was obtained by the slope of the Ln (OD_720_) in the early exponential phase of each growth curve. The doubling time was calculated as Ln2/K′.

### Experimental Evolution.

For the evolution experiment, a single colony of PCC 7942 was grown in liquid BG11 medium in 250 mL glass tubes with a continuous light flux of 1,313 µmol photons m^−2^ s^−1^ at 41 °C and 5% of CO_2_. A control population was grown in parallel, at 25 °C, atmospheric CO_2_ levels and a continuous light flux of 65 µmol photons m^−2^ s^−1^. Cells were grown under serial passage in 200 mL of BG11 supplemented with 10 mM sodium bicarbonate. Iterations started from cultures at OD_720_ = 0.01 which were left to grow for 48 h. At the end of the iteration the OD_720_ was measured, and appropriate dilutions were performed to restart growth at OD_720_ = 0.01. The number of generations in each iteration *n* was calculated as *n* = Log_2_(OD_f_) - Log_2_(OD_i_) where OD_f_ and OD_i_ correspond to the final and initial OD_720_ of the iteration, respectively. Contamination was ruled out in each iteration by plating 100 μL of the culture on LB agar plates and letting them grow at 37 °C for 48 h. Whenever contamination was detected, the culture was discarded, and the LTE was resumed from the previous, uncontaminated batch. Whole genome sequencing was performed at generations 0, 824 (37 passages), and 1,284 (54 passages) to track the emergence of mutations during the experiment.

### Genetic Constructions.

Genetic engineering in PCC 7942 was performed through natural transformation (31). For each transformation, approximately 4 × 10^9^ cells, equivalent to 10 μg of chlorophyll, were mixed with 500 ng of DNA, and incubated in the dark for 16 h at 30 °C and 100 rpm. Transformation mixtures were then deposited onto a 0.45 μm nitrocellulose filter (Millipore) and incubated for 24 h on top of BG11 plates supplemented with appropriate antibiotics, at 30 °C and continuous light. The filter was transferred to new plates with antibiotics every 24 to 72 h. Transformation colonies appeared after 7 to 14 d. Segregation of mutants was achieved by repeatedly streaking individual transformant colonies on selective plates. Mutant genotypes were confirmed by PCR and DNA sequencing using specific primers.

The gene replacement method used to insert the mutations from C11 in the wt strain was based on Matsuoka et al. (59). Briefly, a streptomycin resistant wt strain (MSM1) was obtained by naturally transforming PCC 7942 with the K43R recessive allele of the ribosomal protein S12 (*rps12*-R43) and selection of SmR resistant transformants. This SmR strain was used for the introduction of the individual mutations of C11 in the wt background. A set of two plasmids was designed to insert each mutation. The first one contained a kanamycin/neomycin resistance cassette and the *Synechocystis* sp. PCC 6803 wt version of the *rps12* gene cloned under the promoter of PCC 7942 *psbA1* gene. This gene was flanked by the regions immediately downstream or upstream the gene of interest. Transformants were selected on Neo25 plates and confirmed by their Neo^r^/Sm^s^ phenotype and PCR. To introduce the desired mutation, a second plasmid containing the mutated gene of interest and its flanking regions was introduced through natural transformation. These transformants were then selected through their Neo^s^/Sm^r^ phenotype. Transformants were checked through PCR and Sanger sequencing using appropriate primers. The complete list of mutant strains and plasmids used for their construction can be observed in Supporting information (*SI Appendix*, Tables S2 and S3).

### RNA-Seq Analysis.

Total RNA extraction protocol was an adaptation from Hein et al. (60). 30 mL of a culture at OD_720_ ~ 0.6 were collected by centrifugation at 4,000×*g* for 10 min at RT. The pellet was resuspended in 1.5 mL of Trizol (Ambion-USA) and the suspension was divided in two precooled RNAse-free Eppendorf tubes and immediately frozen in liquid nitrogen and stored at −80 °C. To extract the RNA, the samples were incubated at 65 °C for 15 min, being vortexed several times in the process. 525 µL of chloroform:isoamiloalcohol (24:1) were added. After a 10 min incubation at RT, mixing gently several times, the samples were centrifuged at 6,000×*g*, 3 min at RT. The aqueous phase (470 µL) was transferred to new RNAse-free tubes and 470 µL of chloroform:isoamiloalcohol (24:1) were added, repeating the previous steps. The aqueous phase (370 µL) was transferred again to a new tube, mixed with the other half it was previously separated from. One volume (740 µL) of isopropanol was added, gently mixed, and left precipitating overnight at −80 °C. The RNA was pulled down by 12,000×*g* centrifugation at 4 °C, for 30 min. The pellet was washed three times with 200 µL of ethanol 75% and left to dry. The clean RNA was resuspended in 54 µL of RNAse-free distilled water. 2 µL per lane were loaded in a 2% agarose gel to value sample’s integrity, and 2 µL were used to measure purity and concentration in a Nanodrop (Thermo). The remaining 50 µL were subjected to a DNAse digestion protocol (Invitrogen) and an additional cleaning step running the final volume through an RNA extraction kit column (Qiagen).

The sequencing reads from each sample were trimmed for Illumina adapters remnants and low-quality regions (<Q25) using Trim-Galore (v0.6.2) (61). Surviving high-quality reads were posteriorly aligned against three bacterial rRNA databases (5S, 16S and 23S) from SILVA (https://www.arb-silva.de/) and Rfam (https://rfam.org/) with SortMeRNA to filter out rRNA fragments (v4.3.6) (62). The processed reads were aligned against the complete genome sequence of *S. elongatus*, strain PCC 7942 (GenBank accession number: GCF_000012525.1) using Hisat (v2.2.1) (63), processing the intermediate alignment files and merging the technical replicates with Samtools (v1.6) (64). Individual sample gene expression levels were quantified with featureCounts (v2.0.1) (65). Differential expression of mRNA transcripts was computed with DESeq2 (v1.34.0) (66), using a *P*adj value < 10^−8^ as statistical cutoff. In parallel, normalized expression values were calculated using StringTie (v2.2.1) (67) in Transcripts Per Million (TPMs).

### Statistical Analysis.

Statistical analyses were performed using R-Studio (version 2023.03.0 Build 386, R version 4.2.1) and GraphPad Prism (version 8.3.0). Shapiro–Wilk normality test, followed by a one-way ANOVA and Tukey’s multiple comparison test were used to determine similarities between each strain growth rate for each condition tested. Growth rates grouped by the same letter do not differ statistically with a *P*-value > 0.05. Unpaired Student’s *t* test was used to evaluate differences between two strains, wt and C11, in relation to transcriptomic and metabolic data.

### Additional Materials and Methods.

*SI Appendix*, *SI Methods*, details materials and methods for the determination of pigment and glycogen content, as well as protocols and procedures employed in metabolomic analyses and time-lapse microscopy.

## Supplementary Material

Appendix 01 (PDF)

Dataset S01 (XLSX)

Dataset S02 (XLSX)

Dataset S03 (XLSX)

Dataset S04 (XLSX)

Movie S1. Time-lapse of fluorescence microscopy in Wt_PsigC-YFP.

Movie S2. Time-lapse of fluorescence microscopy in C11_PsigC-YFP.

Movie S3. Time-lapse of fluorescence microscopy in sasAΔ30_PsigC-YFP.

Movie S4. Time-lapse of fluorescence microscopy in ParoK_PsigC-YFP.

## Data Availability

All study data are included in the article and/or supporting information.
