# Supplementary material for: Mutations in the circadian cycle drive adaptive plasticity in cyanobacteria
Source: Proc Natl Acad Sci U S A. 2025 Sep 3;122(36):e2506928122. doi: 10.1073/pnas.2506928122 (PMC12435244; doi:10.1073/pnas.2506928122)
Supplement: Supplementary file 1 — Appendix 01 (PDF) [file pnas.2506928122.sapp.pdf]

PNAS

Supporting Information for

Mutations in the circadian cycle drive adaptive plasticity in cyanobacteria

Alfonso Mendaña[1], María Santos-Merino[1], Raquel Gutiérrez-Lanza, Marina Domínguez-Quintero, Juan Manuel Medina, Ana González-Guerra, Víctor Campa, Miguel Baez, Magaly Ducos-Galand, Rocío López-Igual, Daniel C. Volke, Muriel Gugger, Pablo I. Nikel, Didier Mazel, Fernando de la Cruz and Raúl Fernández-López*

[1]AM and MSM contributed equally to this work

*Corresponding author: Raúl Fernández-López

Email: raul.fernandez@unican.es

This PDF file includes:

SI Methods

SI References

Figures S1 to S12

Tables S1 to S3

Legends for Movies S1 to S4

Legends for Datasets S1 to S4

Other supporting materials for this manuscript include the following:

Movies S1 to S4

Datasets S1 to S4

Supporting Information Methods

Determination of Pigment Content

The extraction of total pigments was performed following the 100% methanol (v/v) method (1). A 1 mL sample of the culture at an OD_{720} of 0.65 was centrifuged at 10,000 *xg*, 5 min and 4°C. The pellet was resuspended in 1 mL of 100% methanol, and the mixture was left at -20°C for 1 hour. After this time, the sample was centrifuged at 10,000 *xg*, 10 min and 4°C, and the absorbance of the supernatant was measured at 652 nm and 665 nm. The chlorophyll *a* (Chl*a*) concentration was calculated using the following equation: Chl*a* (µg mL^{-1}) = 16.29 · Abs_{665} – 8.54 · Abs_{652}, where Abs_{665} and Abs_{652} represent absorbance at 665 nm and 652 nm, respectively.

To measure carotenoid content, the supernatant obtained was used, and its absorbance was measured at 470 nm. The concentration of this pigment was calculated using the following equation: Ct (µg mL^{-1}) = [(1,000 · Abs_{470}) - (2.86 · Chl*a*)]/245, where Abs_{470} corresponds to the absorbance at 470 nm.

The extraction of phycobiliproteins was performed using sonication on ice. A 5 mL sample of the culture at an OD_{720} of 0.65 was pulled down for 5 min at 4,000 xg, RT. The pellet was resuspended in 5 mL of 0.01 M PBS at pH 7.4, and the mixture was sonicated for 5 min. Afterwards, the sample was centrifuged at 4,000 *xg* for 10 min, and the absorbance of the supernatant was measured at 615 nm and 652 nm. The concentration of each pigment was calculated using the following equations, where Abs_{615} and Abs_{652} represent absorbance at 615 nm and 652 nm, respectively: PC (mg mL^{-1}) = ((Abs_{615} – 0.474 · Abs_{652}))/5.34; APC (mg mL^{-1}) = ((Abs_{652} – 0.208 · Abs_{615}))/5.09; Total phycobiliproteins (mg mL^{-1}) = PC + APC.

Cell counting

The relationship between cell density and OD_{720} in different mutant strains was determined by cell counting. For this, 2 mL of culture were sampled: 1 mL was used to measure OD_{720}, and the remaining 1 mL was prepared for cell counting. If the culture was older, the sample was diluted before counting. Three 10 µL replicates were placed in the central area of a Neubauer chamber and allowed to settle for 30 min. Once the cells had settled, those within the 25 central squares were counted under a light microscope.

Determination of intracellular glycogen content

Glycogen content was determined as described previously with minor modifications (2). 2 mL of cyanobacterial cultures were pelleted down by centrifuging at 5,000 xg for 10 minutes. Pellets were flash-frozen in liquid nitrogen and were stored at -80 °C until extraction. For isolation of glycogen, the pellets were resuspended in 200 µL 30% (w/v) KOH and incubated in a heat block at 95 °C for 2 h. Samples were cooled down on ice. Complete precipitation of glycogen was achieved by the addition of 600 µl of cold absolute ethanol and overnight incubation at −20 °C. The precipitated glycogen was recovered by centrifugation at 17,000 xg for 15 min at 4 °C. The supernatant was removed, and the glycogen pellets were dried for 40 min at 60°C using a SpeedVac. The precipitated glycogen was resuspended in 200 µL of miliQ H_2O by vortexing. The homogeneous samples were quantified using the EnzyChrome glycogen assay kit (BioAssay Systems, E2GN-100) according to the manufacturer's instructions.

Darkness-induced lethality assays

The dark-induced lethality phenotypes were determined by measuring the number of viable cells surviving a pulse of total darkness. For this purpose, cells were grown under continuous illumination and ambient air until reaching OD_{720} of 0.4-0.6. At this moment, the light was shut down for a variable time (0, 10, 60, 120 or 1000 min). The survival rate of the culture after darkness was

calculated by counting the number of viable cells on BG11 media obtained immediately before and after the pulse of darkness.

Time-lapse microscopy

YFP-LVA cassettes transcriptionally fused to P_{sigC} or P_{psbA1} promoters were introduced in the neutral site 1 (NS1) of each of our strains through homologous recombination. These cassettes, taken from Martins et al (3), contained a spectinomycin resistance gene for selection. To introduce the construction in C11, which has lost its natural transformation ability due to the *pilA* mutation, a mobilizable plasmid was constructed to shuttle the cassette by conjugation. For this purpose, a RP4 oriT was introduced in plasmid pLA35 from Martins et al (3), which contained the P_{sigC}-YFP-LVA construction. This allowed the mobilization of the cassette to C11 from a MDFpir strain of *E. coli*, using the protocol described by Encinas et al (4). In the rest of the strains, the cassette was introduced through natural transformation following the procedure described above.

Time-lapse images were taken using a Nikon Eclipse Ti2 microscope. For this purpose, cells were grown in liquid BG11 for 2-3 days in 12 h light/12 h darkness conditions to synchronize the population. A 10 µL sample was loaded on a solid BG11-agarose (1.5%) pad and entrained in the microscope for another day under ambient air. Illumination was achieved using the transmitted LED source under Khöler illumination at 387–775 PAR luxes. Light intensity was adjusted using a PM100A light power meter equipped with a slide-type probe. The correlation between PAR lux intensity and µmol photons m^{-2} s^{-1} was calibrated using a LI-250A PAR-light meter (LI-COR®). After entrainment, the cells were kept in continuous illumination for 72 h. Brightfield, red fluorescence and yellow fluorescence images were taken every 45 min under a 60x/1.4 NA PLAN apochromat objective and an ORCA Flash4.0 (Hamamatsu®) camera with a 2x2 binning to reduce background noise. Green and red fluorescence was captured using 472/30 nm excitation – 520/35 nm emission and 578/21 nm excitation – 641/75 nm emission filter cubes (Semrock®) respectively. Time-lapses were simultaneously recorded at five different positions separated at least two fields of view and disposed as the five faces of a dice array. Between different timepoints, transmitted illumination was maintained at the central position with the condenser´s field diaphragm fully open, thus assuring equal light intensity during transmitted illumination for the different positions without cross-illuminating. During the duration of the experiment, cells were maintained in focus using Nikon´s Perfect Focus System, which was let to stabilize for at least 10 s at each position before image acquisition. Whenever a snapshot was found to be out-of-focus it was manually corrected before the next iteration. To maintain transmitted illumination ON during timepoints and stage movements, we took advantage of the microscope software´s (NIS elements) scripting capacity. The resulting fluorescent images were manually segmented with the software Oufti to determine the amount of green and red fluorescence present in the tracked cells along the time-lapse (5). The phase and amplitude of the circadian fluctuations observed through fluorescence microscopy was extracted using Fourier analysis in Matlab (R2024b) and correspond to the average of 5-10 individual trajectories (Supporting Figures S9, S10 and S11).

Metabolomics preparation and analysis

The protocol followed for metabolome extraction was adapted from the one described by Prasannan et al (6). Briefly, a volume of 20 mL with an OD_{720} of 0.6 was withdrawn from the culture grown in the desired conditions and vacuum filtered through a Durapore® 0.45 µm PVDF membrane (Merck Millipore-Ireland). The filter with the biomass was immediately inverted into a petri dish with 1.6 mL of pre-cooled methanol for quenching and incubated at -80°C for an hour, followed by rubbing of the filter against the container to aid cell lysis. Subsequently, the solution was transferred to a reaction tube, and the filter was extracted with 2.4 mL of chloroform. Both fractions were merged and vortexed for 20 min (30 s on, 1 min off, in ice) to disrupt cells. After addition of 2 mL of H_2O, the mixture was vortexed for 10 min. After centrifugation (3,200 *xg*, 15 min, 4°C), two aliquots (750 µL each) of the transparent upper phase were collected and vacuum dried for 8 h. Samples were stored at -20˚C. Upon analysis, samples were resuspended in 180 µL

of H_2O and mixed with 20 µL of internal standard and insoluble particles were removed by centrifugation (17,000 *xg*, 10 min, RT). The internal standard consisted of extract from *E. coli* grown on U-^{13}C-glucose. Metabolites were quantified using LC-MS/MS according to the method of McCloskey et al (7) and the chromatograms were analyzed using the MultiQuant™ software (Sciex, CA, USA). Metabolites were quantified against calibration curves of authentic commercial standards using the internal standard.

Statistical analysis

Doubling time data were analyzed using a two-way ANOVA considering strain, condition, and their interaction as fixed factors. Post-hoc comparisons were performed with estimated marginal means (emmeans), using the wild-type strain under LL, LT conditions as the reference level. Main effects plots for strain and condition, as well as the interaction plot, were generated to visualize the estimated means and interpret the factorial structure of the data. Missing values (n = 3) were handled by type II sum of squares without imputation.

Supporting Information References

1. D. I. Arnon, B. D. McSwain, H. Y. Tsujimoto, K. Wada, Photochemical activity and components of membrane preparations from blue-green algae. I. Coexistence of two photosystems in relation to chlorophyll a and removal of phycocyanin. *Biochim Biophys Acta* **357**, 231–245 (1974).

2. M. Gründel, R. Scheunemann, W. Lockau, Y. Zilliges, Impaired glycogen synthesis causes metabolic overflow reactions and affects stress responses in the cyanobacterium Synechocystis sp. PCC 6803. *Microbiology (Reading)* **158**, 3032–3043 (2012).

3. B. M. Martins, A. K. Das, L. Antunes, J. C. Locke, Frequency doubling in the cyanobacterial circadian clock. *Mol Syst Biol* **12**, 896 (2016).

4. D. Encinas, *et al.*, Plasmid Conjugation from Proteobacteria as Evidence for the Origin of Xenologous Genes in Cyanobacteria. *J Bacteriol* **196**, 1551–1559 (2014).

5. A. Paintdakhi, *et al.*, Oufti: an integrated software package for high-accuracy, high-throughput quantitative microscopy analysis. *Mol Microbiol* **99**, 767–777 (2016).

6. C. B. Prasannan, D. Jaiswal, R. Davis, P. P. Wangikar, An improved method for extraction of polar and charged metabolites from cyanobacteria. *PLoS ONE* **13**, e0204273 (2018).

7. D. McCloskey, J. D. Young, S. Xu, B. O. Palsson, A. M. Feist, MID Max: LC–MS/MS Method for Measuring the Precursor and Product Mass Isotopomer Distributions of Metabolic Intermediates and Cofactors for Metabolic Flux Analysis Applications. *Anal. Chem.* **88**, 1362–1370 (2016).

8. M. Matsuoka, K. Takahama, T. Ogawa, Gene replacement in cyanobacteria mediated by a dominant streptomycin-sensitive rps12 gene that allows selection of mutants free from drug resistance markers. *Microbiology (Reading)* **147**, 2077–2087 (2001).

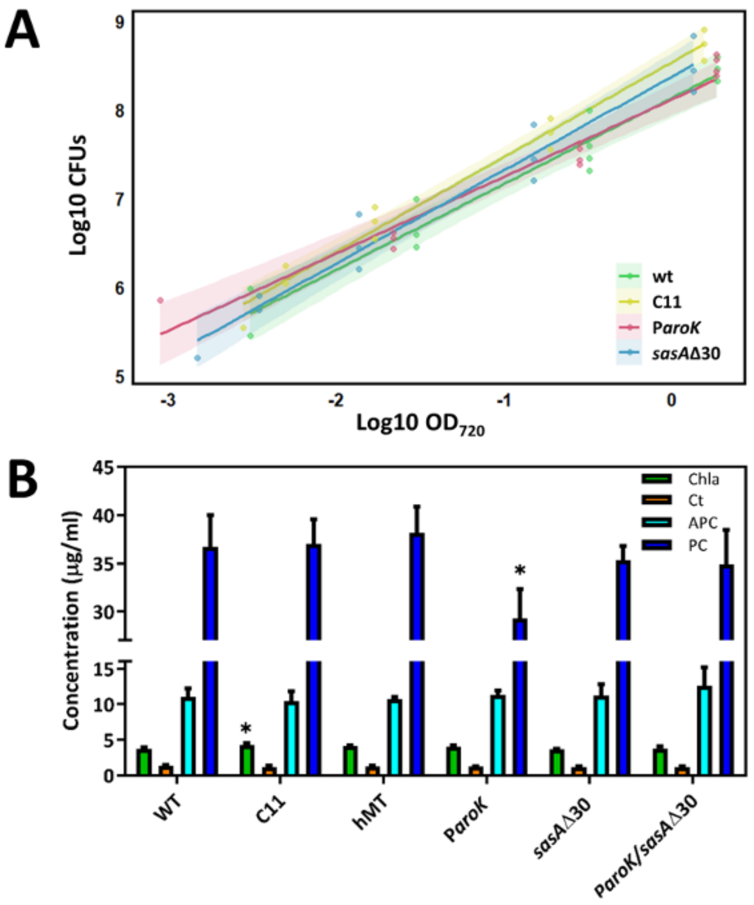

Supporting Figure S1.- Colony forming units (CFUs) per OD_{720} and pigment content in the wt, C11 and the individual mutants. (A) Correlation between the CFUs and the optical density measured at 720 nm in the wt and mutants shown in the legend. Each dot represents a culture of cyanobacteria for which the OD_{720} (x-axis) and the CFUs (y-axis) were measured. Lines and shadowed areas correspond, respectively, to the average and standard deviation of 3 samples measured at the same OD_{720}. (B) Pigment content, expressed as µg mL^{-1}, in the strains shown in the legend. Green: Chl*a*, chlorophyll *a*; Orange: Ct, carotenoids; Cyan: APC, allophycocyanin; Navy: PC, phycocyanin. Bars indicate the mean and standard deviation of at least three independent replicates. *p-value < 0.05, obtained by an ANOVA test followed by a Dunnet's multiple comparison test using the wt strain as a control. All measurements were performed at 30ºC, and under LL and LC conditions.

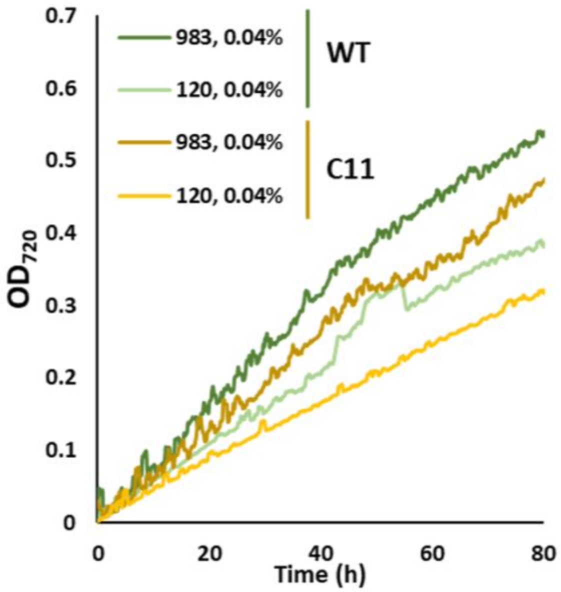

Supporting Figure S2.- The evolved strain grows slower than the *wt* at low CO_2 concentrations. Growth curves corresponding to the wt (green lines) and the evolved strain (yellow lines), measured as OD_{720} (y-axis) across time (x-axis). All measurements were performed in BG11 medium at 41ºC, 0.04% CO_2 and the light intensities shown in the chart (μmol photons m^{-2} s^{-1}).

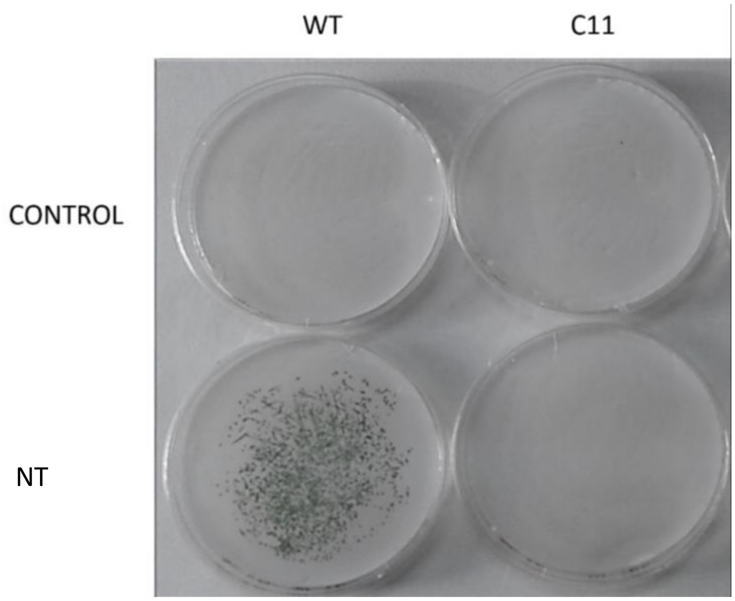

Supporting Figure S3.- Natural transformation of the wt and the evolved strains. Control with no DNA and natural transformation (NT) with the plasmid pMSM2 of the WT (left) and the evolved strain, C11 (right). Experiments were performed at 41ºC, HL and LC conditions.

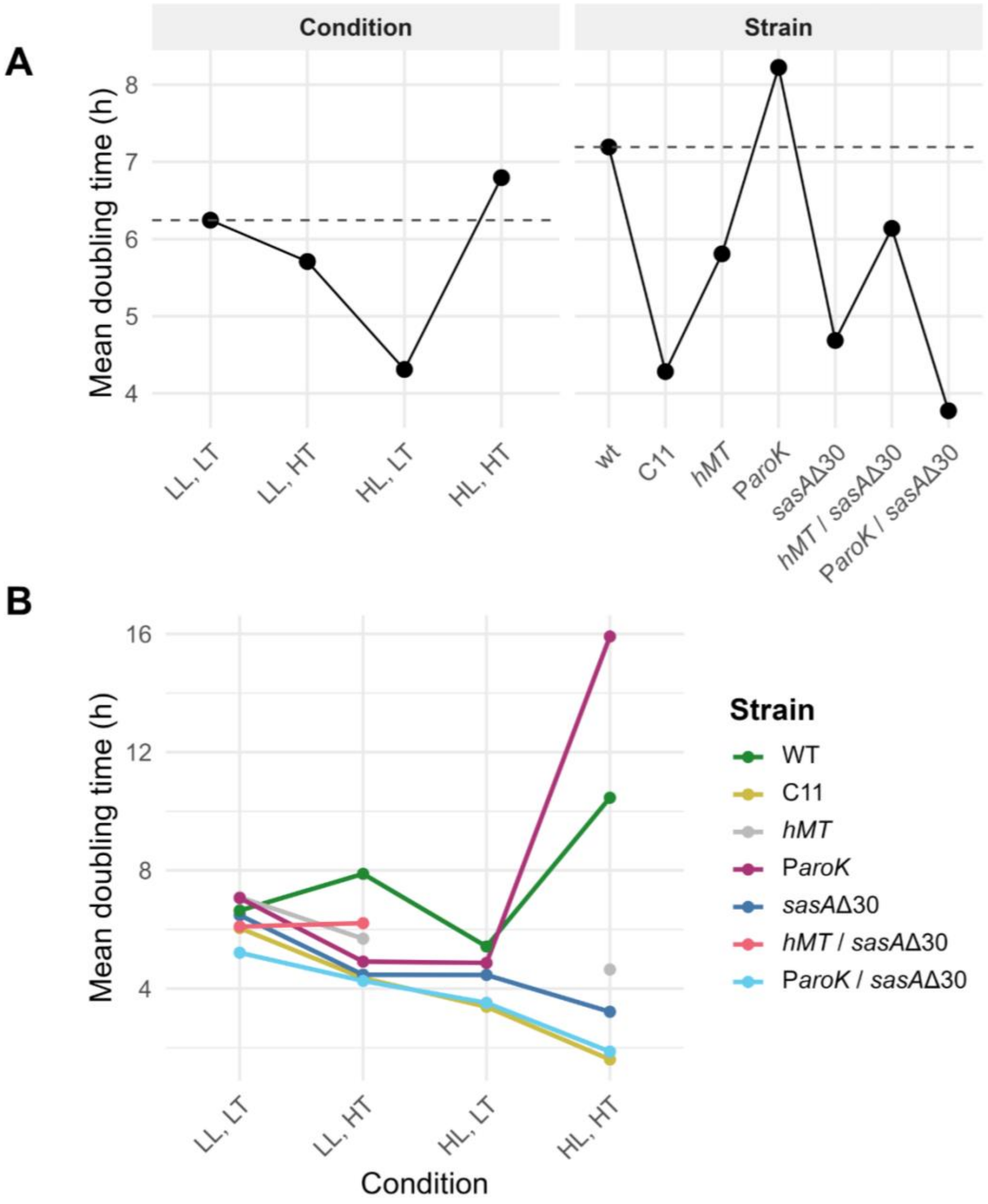

Supporting Figure S4- Two-way ANOVA analysis of the effect of strain and environmental conditions on doubling time. (A) Main effects analysis for Environmental Condition (left panel) and Strain (right panel). Each black dot is the average of all the measurements of doubling time (y-axis) performed at that particular condition or strain. The dashed horizontal line indicates the average of the control condition (LL, LT) or strain (wt). LL = low light, HL = high light, LT = low temperature, HT = high temperature. (B) Interaction analysis of Environmental Condition + Strain on the doubling time. Each dot represents the mean of the doubling time for that particular combination. When two lines are parallel, it indicates that the interaction between Environmental Condition + Strain is not statistically significant, whereas when the lines are not parallel, that particular interaction between strain and condition is statistically significant.

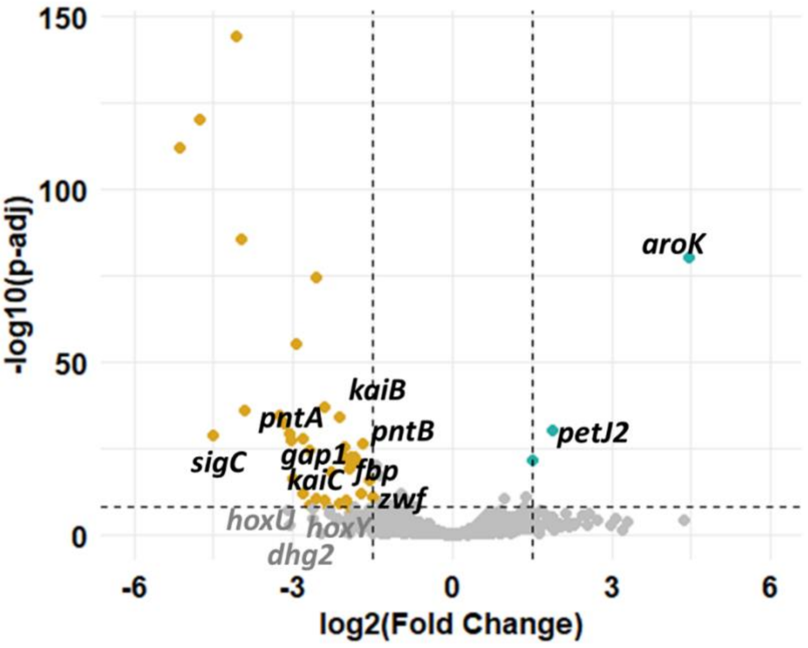

Supporting Figure S5.- A comparison between the transcriptomes of C11 and the wt at HL. Volcano plot showing the changes in gene expression between C11 and the wt under high light intensity (HL, 983 µmol photons m^{-2} s^{-1}), 3% CO_2 (HC) and 30°C. Dots represent the log_2 fold change of individual genes (x-axis) against their adjusted p-value (y-axis). Dots shown in teal correspond to genes with at least 1.5-fold increase with adjusted p-value < 10^{-8}. Dots shown in orange correspond to genes with 1.5-fold decrease in expression with adjusted p-value < 10^{-8}.

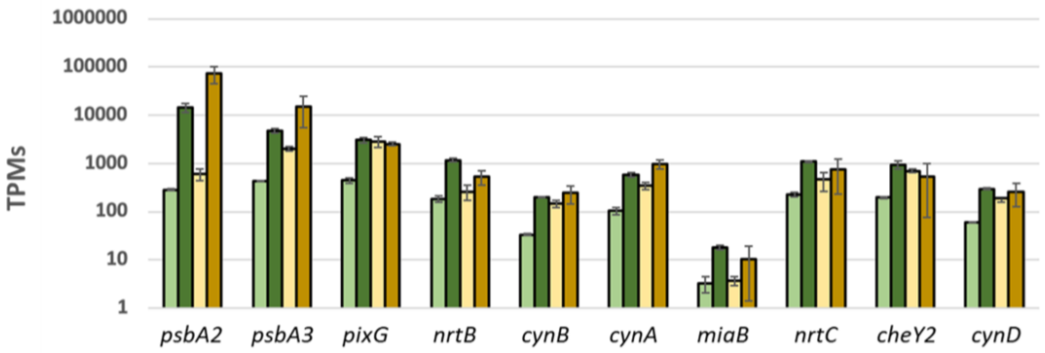

Supporting Figure S6.- Gene expression levels of genes activated during growth in high light. Expression levels are indicated in TPMs (y-axis). Bars represent the average and standard deviation of three separate experiments. The conditions of the experiment were the same as in Figure 2 in the main text (HC and 30ºC). The strains here represented are, from left to right: WT under LL (light green), WT under HL (dark green), C11 under LL (light yellow), C11 under HL (dark yellow).

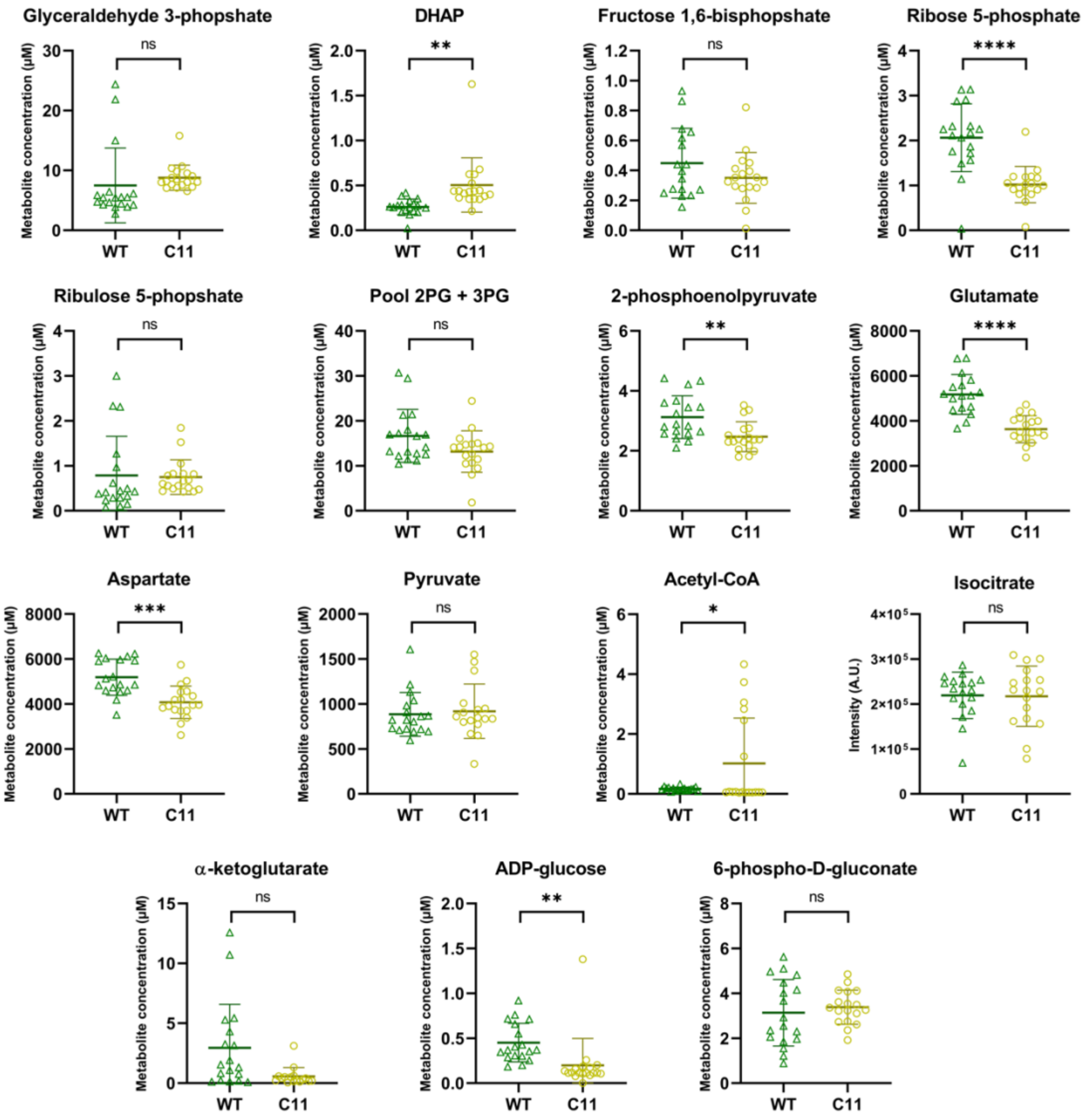

Supporting Figure S7.- Metabolite concentrations in the wt (green) and C11 (yellow) from the central carbon metabolism pathways shown in Figure 3. For those metabolites for which a control standard was available, concentrations are expressed as µM. The rest are shown as arbitrary units (A.U.). Significance shown as ns, non-significant, *p-value < 0.05, **p-value < 0.01, ***p-value < 0.001, ****p-value < 0.0001. Culture conditions were 30ºC, HL and HC.

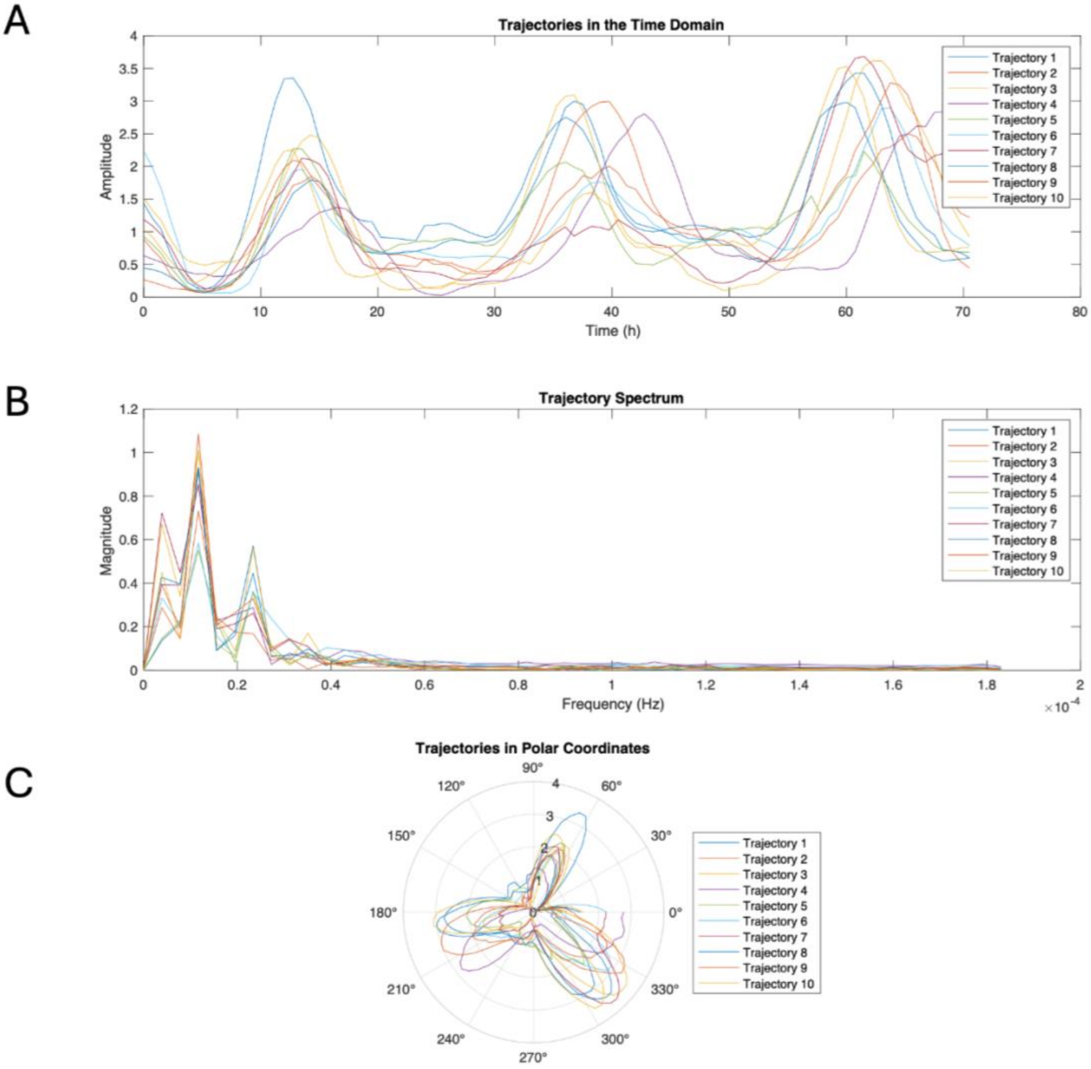

Supporting Figure S8.- Calculation of the period and amplitude of the P_{sigC}-YFP circadian fluctuations in the wt. Data from Figure 4A was used to represent the trajectories in the time domain (A), the Fourier space (B) and the polar coordinates (C) in a time frame of t = 72 h. Each trajectory represents the background-subtracted fluorescence levels of an individual cell. Mean Amplitude: 1.5264 ± 0.23315. Mean Period: 23.7738 ± 0 h.

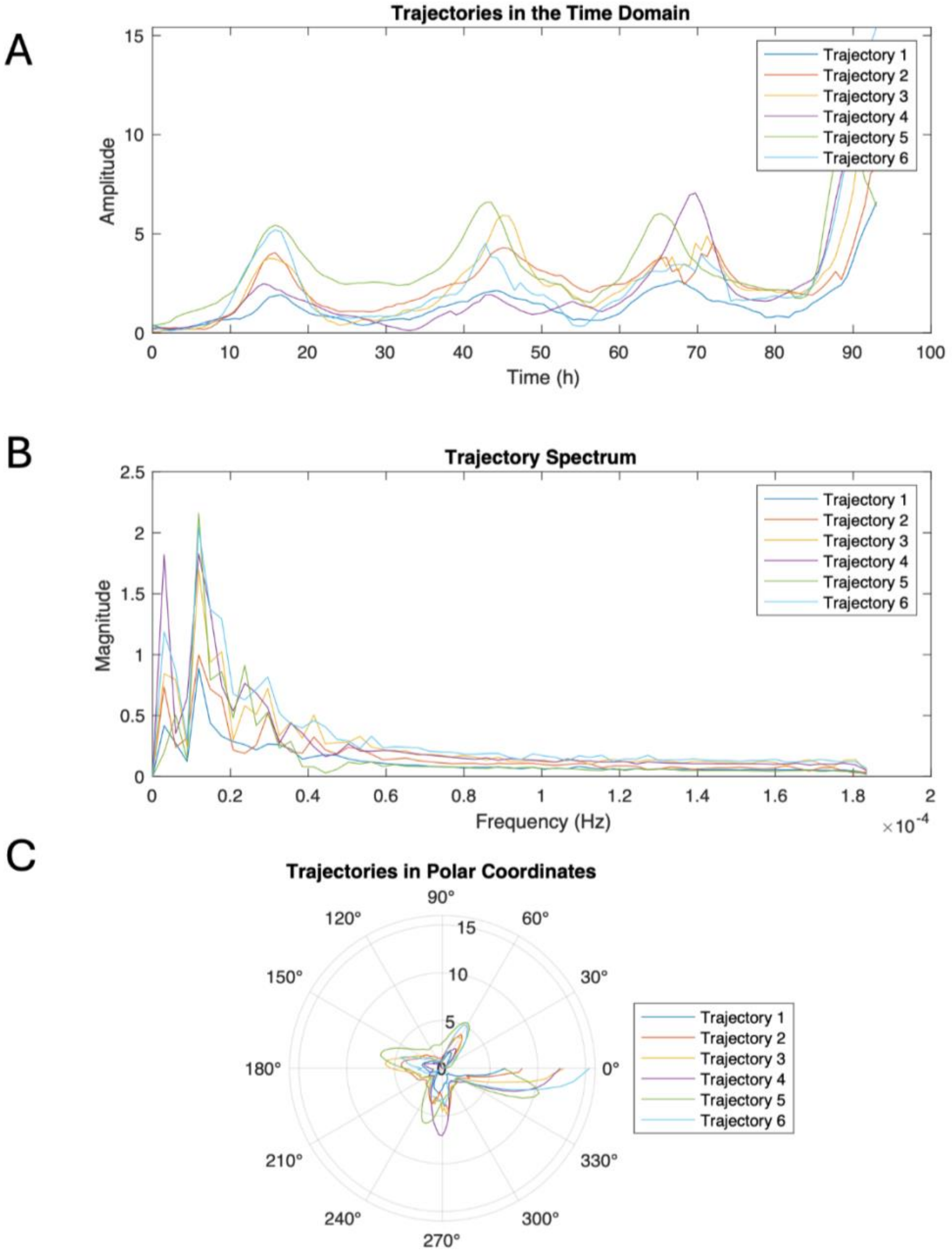

Supporting Figure S9.- Calculation of the period and amplitude of the P_{sigC}-YFP circadian fluctuations in the P_{aroK} mutant. Data from Figure 4D was used to represent the trajectories in the time domain (A), the Fourier space (B) and the polar coordinates (C) in a time frame of t = 72 h. Each trajectory represents the background-subtracted fluorescence levels of an individual cell. Mean Amplitude: 5.4177 ± 1.6186. Mean Period: 23.461 ± 0 h.

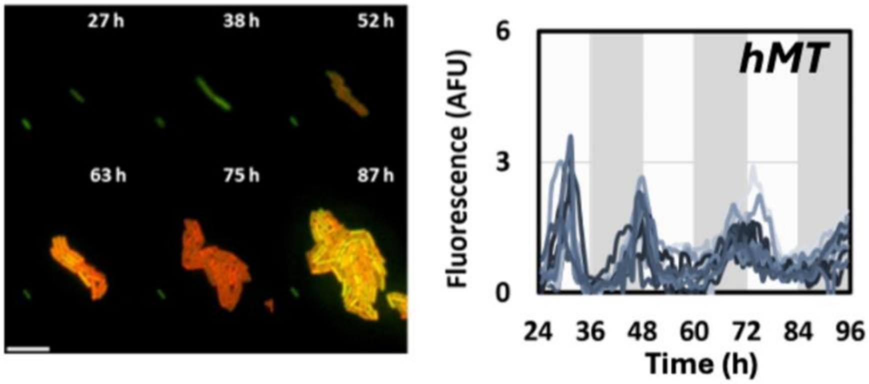

Supporting Figure S10.- Circadian rhythm of *sigC* in the *hMT* mutant. Microphotographs (left) and fluorescence traces (right) obtained from a P_{sigC}_YFP transcriptional fusion in the *hMT* strain. Time-lapse experiments were performed on cultures pre-synchronized after growing for 72 h. in light/dark conditions. Each trace on the graphs represents the values obtained for a single cell, tracked for 72 h. Dark and light areas of the charts indicate, respectively, the periods of subjective night and day. All experiments were performed under LC and LT conditions, and 60 µmol photons m^{-2} s^{-1} when light was used. The scale bar represents 10 µm.

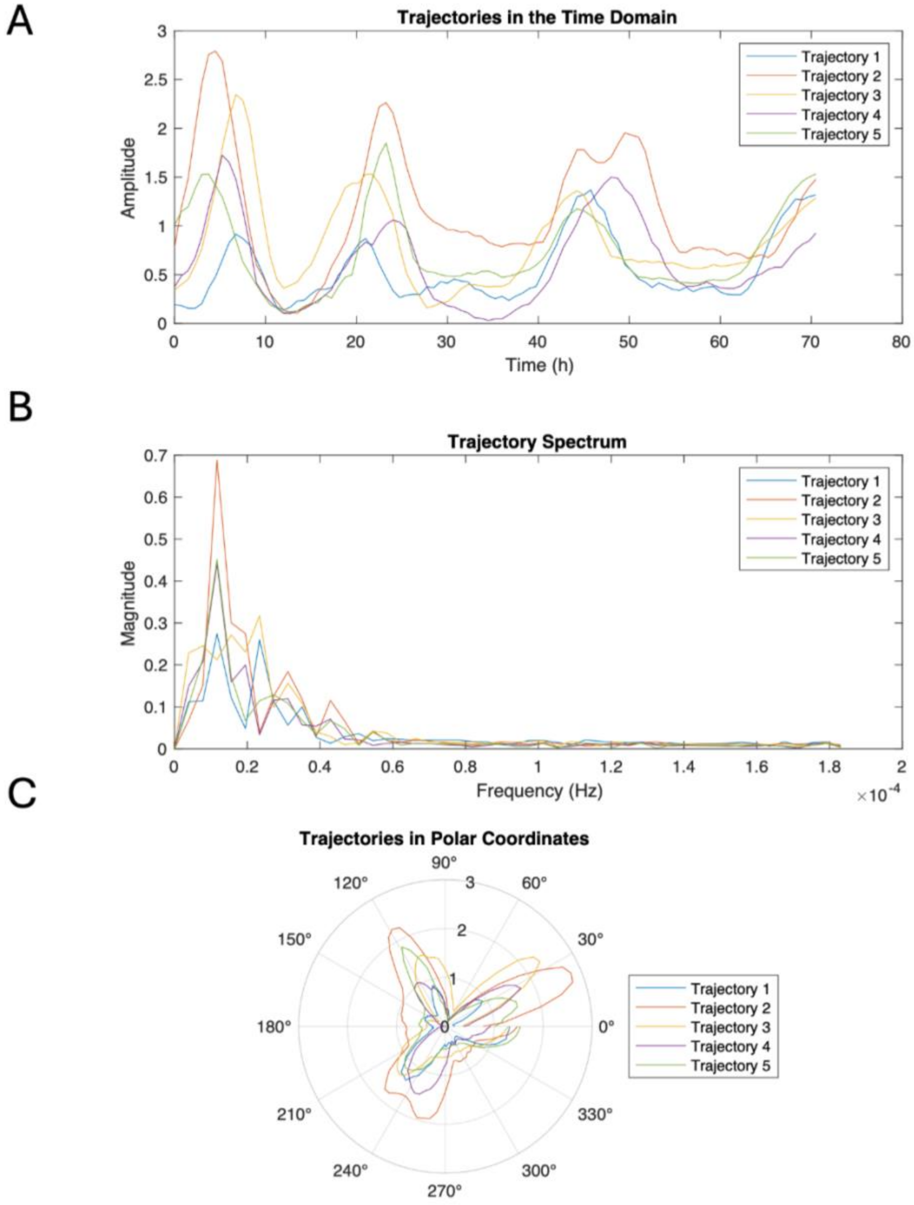

Supporting Figure S11.- Calculation of the period and amplitude of the P_{sigC}-YFP circadian fluctuations in the *hMT* mutant. Data from Supporting Figure S7 was used to represent the trajectories in the time domain (A), the Fourier space (B) and the polar coordinates (C) in a time frame of t = 72 h. Each trajectory represents the background-subtracted fluorescence levels of an individual cell. Mean Amplitude: 0.95303 ± 0.2756. Mean Period: 21.3964 ± 5.316 h.

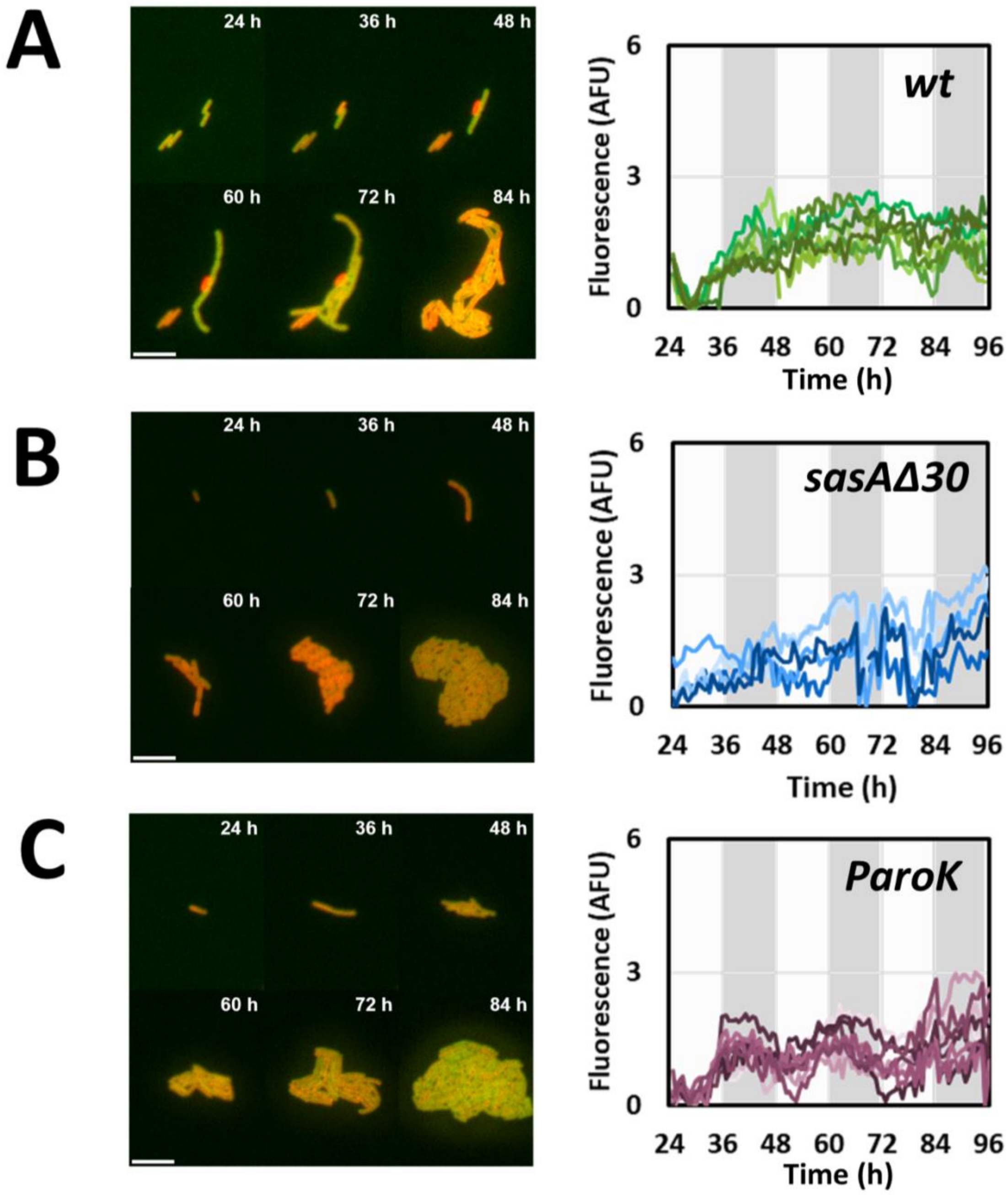

Supporting Figure S12.- Circadian rhythm of *psbA1* in individual mutants.
Microphotographs (left) and fluorescence traces (right) obtained from a P_{psbA1}_YFP transcriptional fusion in each of the strains indicated. Time-lapse experiments were performed on cultures pre-synchronized after growing for 72 h in light/dark conditions. Each trace on the graphs represents the values obtained for a single cell, tracked for 72 h. Dark and light areas of the charts indicate, respectively, the periods of subjective night and day. All experiments were performed under LC and LT conditions, and 30 µmol photons m^{-2} s^{-1} when light was used. The scale bar represents 10 µm.

Table S1. Two-way ANOVA analysis results of the doubling times of the strains and the conditions used in Figure 1B in this study. Asterisks indicate (* p.value<0.05; ** p.value<0.01; *** p.value<0.001)

Strain, Conditions & Interactions	Estimate	Std.error	Statistic	p.value	significance
(Intercept)	6.6400745	0.38719663	17.149102	2.05E-36	***
C11	-0.59257173	0.4998687	-1.18545477	0.23781769	
hMT	0.45058714	0.67064424	0.67187208	0.50275678	
P*aroK*	0.42416982	0.67064424	0.63248112	0.52808965	
sasAΔ30	-0.1564574	0.54757873	-0.28572586	0.77550448	
hMT/sasAΔ30	-0.53999646	0.54757873	-0.9861531	0.32573532	
P*aroK/sasAΔ30*	-1.42492501	0.54757873	-2.60222858	0.01024421	*
LL+HT	1.24280291	0.54757873	2.26963329	0.02473607	*
HL+LT	-1.21664831	0.54757873	-2.22186921	0.0278744	*
HL+HT	3.81753168	0.67064424	5.69233503	6.92E-08	***
C11:LL+HT	-2.93372451	0.83644032	-3.50739251	0.00060626	***
hMT:LL+HT	-2.6449697	0.94843417	-2.7887752	0.0060168	**
P*aroK*:LL+HT	-3.39284281	0.94843417	-3.57730974	0.00047529	***
sasAΔ30:LL+HT	-3.25565307	0.86579799	-3.76029181	0.00024733	***
hMT/sasAΔ30:LL+HT	-1.12923008	0.86579799	-1.30426508	0.19425333	
P*aroK/sasAΔ30*:LL+HT	-2.19687294	0.86579799	-2.53739669	0.01224781	*
C11:HL+LT	-1.44485783	0.7069211	-2.04387426	0.04281433	*
P*aroK*:HL+LT	-0.97862581	0.8318318	-1.17647078	0.24137524	
sasAΔ30:HL+LT	-0.80358798	0.77439326	-1.03770012	0.30117349	
P*aroK/sasAΔ30*:HL+LT	-0.47931225	0.77439326	-0.61895199	0.53693969	
C11:HL+HT	-8.262004	0.92171234	-8.96375543	1.64E-15	***
hMT:HL+HT	-6.26291184	1.02442599	-6.11358154	8.91E-09	***
P*aroK*:HL+HT	5.02902774	0.94843417	5.30245311	4.28E-07	***
sasAΔ30:HL+HT	-7.08423808	0.86579799	-8.18232219	1.43E-13	***
hMT/sasAΔ30:HL+HT	NA	NA	NA	NA	
P*aroK/sasAΔ30*:HL+HT	-7.16269988	0.89419232	-8.0102454	3.76E-13	***

Table S2. Synel PCC7942 strains used in this work

Name	Genotype	Plasmids used to generate this strain	Antibiotic resistance	Reference
**Se7942**	*Synechococcus elongatus* PCC7942 wild-type strain obtained from the PCC.	-	-	Pasteur Culture Collection
**GRPS1**	Se7942 with *rps12* R43 mutation	-	SmR	(8)
**C11**	Experimentally evolved Se7942 with mutations described in Figure 1	-	-	This work
**MSM1 (WT)**	Se7942 with *rps12* R43 mutation	pMSM2	SmR	This work
hMT	MSM1 with C11's R71* mutation on Synpcc7942_0329	pAMG1 and pAMG21	SmR	This work
**P*aroK***	MSM1 with C11's C->T mutation on Synpcc7942_0894 promoter region	pMSM22 and pMSM24	SmR	This work
***sasA*Δ30**	MSM1 with C11's Q352* mutation on Synpcc7942_2114	pMSM80 and pMSM82	SmR	This work
hMT*/P*aroK	P*aroK* strain with C11's R71* mutation on Synpcc7942_0329 and the construction *rps12*-Km downstream	pAMG16	NeoR	This work
***hMT*/ *sasA*Δ30**	*sasA*Δ30 strain with C11's R71* mutation on Synpcc7942_0329 and the construction *rps12*-Km downstream	pAMG16	NeoR	This work
**P*aroK*/ *sasA*Δ30**	MSM9 with the WT version of Synpcc7942_0918	pMSM22	NeoR	This work
**MSM1 (wt) P*sigC***	MSM1 with an SpR+Promoter of *sigC*+YFP+LVA tag cassette inserted in the NSI	pMSM321	SmR SpR	This work
**C11 P*sigC***	C11 with an SpR+Promoter of *sigC*+ YFP+LVA tag cassette inserted in the NSI	pMSM321	SpR	This work
hMT* P*sigC	*hMT* with an SpR+Promoter of *sigC*+ YFP+LVA tag cassette inserted in the NSI	pMSM321	SmR SpR	This work
**P*aroK* P*sigC***	P*aroK* with an SpR+Promoter of *sigC*+ YFP+LVA tag cassette inserted in the NSI	pMSM321	SmR SpR	This work
sasA*Δ30 P*sigC	*sasA*Δ30 with an SpR+Promoter of *sigC*+ YFP+LVA tag cassette inserted in the NSI	pMSM321	SmR SpR	This work
**P*aroK* P*psbA*1**	P*aroK* with an SpR+Promoter of *psbA*1+ YFP+LVA tag cassette inserted in the NSI	pMSM279	SmR SpR	This work
***sasA*Δ30 P*psbA*1**	*sasA*Δ30 with an SpR+Promoter of *psbA*1+ YFP+LVA tag cassette inserted in the NSI	pMSM279	SmR SpR	This work
**Δ*rpaA***	MSM1 with a deletion on Synpcc7942_0095	pMSU_0095	SmR NeoR	This work

Table S3. Plasmids used in this work

Plasmid	Characteristics	Construction	Source or reference
**pLA35**	P*psbA1*::YFP_LVA in pAM2314	-	(3)
**pAD03**	P*sigC*::YFP_LVA in pAM2314	-	(3)
**pSB1C3**	Biobrick cloning vector	-	http://parts.igem.org/Part:pSB1K3
**pDEP52**	pSB1C3::(*rps12-nptI*)	-	David Encinas Pisa thesis (2014)
**pMSU_0095**	Vector containing a truncated version of Synpcc7942_0095 with a kanamycin resistance gene (*nptI*)	-	Danny Ducat Lab
**pMSM2**	pSB1C3 with an 1896 pb region from *SyneI* PCC7942 GRPS1 containing *rps12*-R43	Biobrick method. Oligos 1 and 2 used for insert amplification, cloned with EcoRI and PstI	This work
**pMSM18**	pDEP52 with a 501 pb region downstream from *aroK* (Synpcc7942_0894) cloned to the right of *rps12-nptI*	Isothermal Assembly. Oligos 3 and 4 for insert amplification, 5 and 6 for vector amplification	This work
**pMSM22**	pMSM18 with the mutated P*aroK* (Synpcc7942_0894) from C11 cloned to the left of *rps12-nptI*	Isothermal Assembly. Oligos 7 and 8 for insert amplification, 9 and 10 for vector amplification	This work
**pMSM24**	pSB1C3 with the mutated P*aroK* (Synpcc7942_0894) from C11 and its downstream region	Isothermal Assembly. Oligos 7 and 4 for insert amplification, 5 and 10 for vector amplification	This work
**pMSM76**	pDEP52 with the mutated *sasA* (Synpcc7942_2114) from C11 cloned to the right of *rps12-nptI*	Isothermal Assembly. Oligos 11 and 12 for insert amplification, 5 and 6 for vector amplification	This work
**pMSM80**	pMSM76 with a 996 pb region upstream of *sasA* (Synpcc7942_2114) cloned to the left of *rps12-nptI*	Isothermal Assembly. Oligos 13 and 14 for insert amplification, 5 and 6 for vector amplification	This work
**pMSM82**	pMSM80 without the *rps12-nptI* region	Isothermal Assembly. Oligos 13 and 15 for insert amplification, 16 and 10 for vector amplification	This work
**pMSM279**	pLA35 with the oriTRP4	OriTRP4 cloned with BamHI and XhoI	This work

**pMSM321**	pMS279 with the P*sigC* from pAD03	P*sigC* cloned with NotI and SmaI	This work
**pAMG16**	pDEP52 with the mutated *hMT* (Synpcc7942_0329) from C11 cloned to the left of *rps12-nptI* and a 543 pb region downstream from *hMT* to the right	Isothermal Assembly. Oligos 17 and 18 for *hMT* amplification, 19 and 20 for rps12-*nptI*, 21 and 22 for downstream region and 23 and 24 for vector amplification	This work
**pAMG21**	pSB1C3 with the mutated *hMT* (Synpcc7942_0329) from C11 and its downstream region	Isothermal Assembly. Oligos 17 and 20 for insert amplification, 23 and 24 for vector amplification	This work

Movie S1 (separate file). Time-lapse of fluorescence microscopy in Wt_PsigC-YFP

Movie S2 (separate file). Time-lapse of fluorescence microscopy in C11_PsigC-YFP

Movie S3 (separate file). Time-lapse of fluorescence microscopy in sasAΔ30_PsigC-YFP

Movie S4 (separate file). Time-lapse of fluorescence microscopy in ParoK_PsigC-YFP

Dataset S1 (separate file). C11 sequencing analysis.

Dataset S2 (separate file). Transcriptomics in continuous light.

Dataset S3 (separate file). Metabolomics in high light

Dataset S4 (separate file). Transcriptomics in circadian light.
